# Exploring the Anticancer Properties of 4-Phenylthiazole-Based Ru(II) and Os(II) Metallacycles Featuring 1-Methylimidazole as *N*-Donor Functionality

**DOI:** 10.1155/bca/6352081

**Published:** 2025-09-18

**Authors:** Paul Getreuer, Theresa Mendrina, Steven van Terwingen, Laura Marretta, Orsolya Dömötör, Dominik Wenisch, Michaela Hejl, Petra Heffeter, Walter Berger, Michael A. Jakupec, Alessio Terenzi, Bernhard K. Keppler, Wolfgang Kandioller

**Affiliations:** ^1^Faculty of Chemistry, Institute of Inorganic Chemistry, University of Vienna, Währinger Str. 42, 1090 Vienna, Austria; ^2^Faculty of Chemistry, Vienna Doctoral School in Chemistry (DoSChem), University of Vienna, Währinger Str. 42, 1090 Vienna, Austria; ^3^Center of Cancer Research and Comprehensive Cancer Center, Medical University of Vienna, Borschkegasse 8a, 1090 Vienna, Austria; ^4^Research Cluster “Translational Cancer Therapy Research”, Währinger Str. 42, 1090 Vienna, Austria; ^5^STEBICEF-Department, University of Palermo, Viale Delle Scienze, Ed. 17, 90128 Palermo, Italy; ^6^Department of Molecular and Analytical Chemistry, Interdisciplinary Excellence Centre, University of Szeged, Dóm tér 7-8, 6720 Szeged, Hungary

**Keywords:** 2D & 3D cytotoxicity, anticancer, *C,N-*chelates, in vivo studies, leaving group variation, metallacycles, metallodrugs

## Abstract

Ten organometallic complexes of the general formula [M(*p*-cymene)thi_CΛN_MeIm]NO_3_ (M = Ru, Os; MeIm = 1-methylimidazole, thi = 4-phenylthiazole) differing in their substituents on the 4-phenylthiazole scaffold were prepared and characterized by standard analytical methods. The antiproliferative activity of the compounds was investigated in human lung adenocarcinoma (A549), colon adenocarcinoma (SW480), and human ovarian teratocarcinoma (CH1/PA-1) cell lines. IC_50_ values were in the low micromolar range with two exceptions. Additionally, the cytotoxicity of selected compounds was determined in the HCT116 colon carcinoma cell line in both 2D (monolayer) and 3D (multicellular spheroid) cultures. For selected compounds, the capacity of ROS induction was investigated in SW480 cells. Cellular accumulation experiments, as well as studies regarding stability and reactivity in aqueous solution, were performed, providing conclusive explanations for the observed differences in cytotoxicity. Furthermore, amino acid and DNA interaction studies were performed to elucidate aspects of the mechanism of action. The obtained insight into the antiproliferative activity in multicellular spheroids compelled us to perform in vivo studies, revealing the unexpected therapeutic efficacy of an in vitro inactive complex.

## 1. Introduction

Platinum(II) chemotherapeutics have been effectively utilized for over 4 decades for the treatment of numerous cancer types [1–6]. However, these platinum agents are lacking selectivity, making rapidly dividing cells, such as those in the bone marrow and gastrointestinal tract, susceptible to adverse effects and leading to the development of severe and dose-limiting side effects, such as nausea, vomiting, nephrotoxicity, neurotoxicity, and loss of high-frequency hearing. Moreover, the prevalence of platinum resistance in the clinic is a significant drawback, especially considering that platinum drugs are applied in over half of all chemotherapy treatments [7–10]. Therefore, there is an urgent need for alternative chemotherapeutics with increased selectivity and novel modes of action to overcome resistance [5, 6].

**Figure 1:**
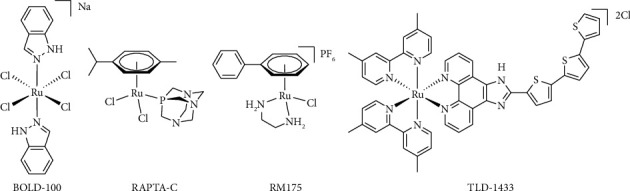
Structures of promising Ru(III) (BOLD-100) and Ru(II) (RAPTA-C, RM175, TLD-1433) anticancer agents.

Due to the chemical similarity within the platinum group metals (Pt, Pd, Rh, Ir, Ru, and Os), ruthenium complexes garnered considerable attention in the last decades [5, 6, 10]. Their development has been stimulated greatly by the first-in-class Ru(III) metallodrug indazolium *trans*-[tetrachlorido-bis(1*H*-indazole) ruthenate(III)] (KP1019), which exhibited significant anticancer activity with mild toxicity in a clinical Phase 1 study [11]. In subsequent studies, its sodium analog BOLD-100 (Figure 1) was investigated and proved superior due to its enhanced aqueous solubility [5, 6]. The latter Ru(III)-based drug features promising activity, especially in gastrointestinal neuroendocrine tumors and non–small-cell lung cancer, successfully completing a clinical Phase 1b/2a trial [12–14]. The proposed pharmacology of BOLD-100 involves rapid binding to blood proteins, accumulation, and reduction in the hypoxic tumor milieu, yielding the highly reactive Ru(II) species. BOLD-100 downregulates GRP78 [15] and disrupts the lipid metabolism [16], among other biological processes. Reductive environments are uncommon in healthy tissue, which allows for a selective activation of the drug in proximity to its cellular targets [5, 6]. Alongside BOLD-100, the Ru(II) complex TLD-1433 (Figure 1) is similarly advanced in clinical development, as it is currently undergoing a Phase 2 trial for its potential use in the photodynamic therapy of non–muscle-invasive bladder cancer [5, 17–19].

The lacking stability of Ru(II) complexes relative to Ru(III) under typical physiological conditions can be circumvented by coordination of a π-bonded arene [20]. This constitutes a class of remarkably stable pseudo-octahedral complexes often referred to as “piano-stool” complexes, where the arene embodies the “seat” and the three ligands the “legs” of the stool [5, 6]. These complexes often feature halide leaving groups, priming the compound for biological interactions via substitution of the halide moiety by a water molecule (activation-by-aquation) [5, 6, 21]. The most investigated representatives of that compound class are Dyson's RAPTA and Sadler's RAED complexes (Figure 1) [6, 22–25].

Given the inherent instability often associated with conventional bidentate ligands with *O,O*; *N,O*; or *S,O* motifs in Ru(II) complexes [26, 27], the utilization of different coordination modes, such as *N,N* or *C,N* ligand scaffolds, has been pursued with promising results [5, 6, 13]. *C,N* bidentate ligands have garnered considerable attention in recent years, due to their enhanced stability under physiological conditions, while showing notable antiproliferative activities [5, 6, 13, 28–34]. Lately, we reported the promising antiproliferative properties of 4-phenylthiazole-derived Ru(II) and Os(II) arene complexes with the general form [M(arene)L_CΛN_Cl]. While these complexes have limited solubility in aqueous environments, their significant in vitro cytotoxicity and the yet unknown mechanism of action provide compelling reasons for further research [5].

As mentioned above, the exchange of the chlorido ligand for a water molecule is assumed to be an essential step in the mechanism of action. However, premature hydrolysis under physiological conditions leads to undesired interactions with biomolecules and thereby potential side effects. Hence, protecting the active cyclometalated 4-phenylthiazole core (in a prodrug fashion) until it reaches the tumor represents a promising approach toward more selective anticancer agents. In that sense, the substitution of the labile chlorido ligand by *N*-donor leaving groups not only enhances the stability of the compounds in physiological environments but also significantly improves their aqueous solubility [13]. This enhancement is ascribed to the higher affinity of the metal center to *N*-donor ligands, thereby limiting premature hydrolysis, in contrast to the parental chlorido complexes.

Tumor microenvironments usually feature notable extracellular acidosis due to enhanced metabolic activity. The utilization of that feature for acid-based activation reflects a common strategy in the development of novel cancer therapeutics. A previous study suggests MeIm as an appropriate monodentate ligand due to its pH-dependent cleavage and the resulting activation of the complex [35, 36]. Thus, we facilitated a leaving group exchange by introducing MeIm as monodentate *N*-donor into Ru(II) and Os(II) complexes bearing cyclometalated 4-phenylthiazole ligands instead of the chlorido moiety. This study presents the synthesis, characterization, and stability assessment of the complexes, alongside an evaluation of their antiproliferative activity in both 2D and 3D cancer cell models. Their anticancer in vivo efficacy in terms of mice's overall survival is presented, accompanied by the organ distribution of the drug. In addition, we explore their mechanism of action, including investigations into reactive oxygen species (ROS) generation, amino acid binding, and interactions with G-quadruplex DNA structures.

## 2. Results and Discussion

### 2.1. Synthesis and Characterization

**Scheme 1:**
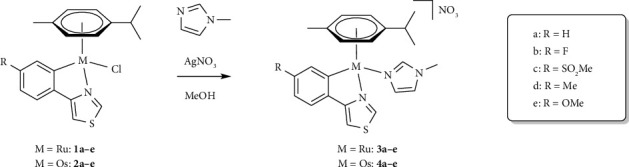
Synthesis of substituted, 4-phenylthiazole-based Ru(II) (**3a–e**) and Os(II) (**4a–e**) metallacycles.

Ten ruthena- (**3a–e**) and osmacycles (**4a–e**) differing in the substituents on the 4-phenylthiazole ligand were synthesized starting from the previously published chlorido complexes (**1a–e**, **2a–e**) [5]. MeIm was introduced as *N*-donor moiety via chlorido ligand abstraction using AgNO_3_ under light exclusion in methanol (Scheme 1). The reaction was complete after 2 h of stirring at ambient temperature according to ^1^H-NMR. Subsequently, purification of the crude product using flash chromatography afforded the target compounds in elemental analysis purity in good to excellent yields (67%–92%). Nitrate was chosen as counterion to improve the solubility of the complexes.

The metallacycles were characterized by standard analytical methods, such as ^1^H-NMR, ^13^C-NMR, 2D-NMR techniques, elemental analysis, high-resolution mass spectrometry (HRMS), and single-crystal X-ray diffraction.

^1^H-NMR and ^13^C-NMR analyses confirmed the incorporation of the MeIm moiety (see Figures S1–10), evidenced by a significant downfield shift of the H2 proton MeIm signal (1.1–1.2 ppm). Compared to the chlorido complexes, the thiazole-2 proton also exhibits a pronounced downfield shift within the same range. Notably, the aromatic protons of *p*-cymene (*p*-cym) are displaced downfield to a similar extent that the aliphatic protons are shifted upfield (0.3–0.4 ppm). The residual proton signals remain within the same range as the parental chlorido complex (±0.1 ppm). ^1^H-NMR spectra of ruthenium and osmium analogs show remarkable similarity. The only significant difference is the distinct separation of the aromatic *p*-cym signals, which coincide in the case of the ruthenium complexes.

Solubility is an important factor for the application of metallodrugs. DMF was used as solubilizer to allow comparison to the respective chlorido complexes where the usage of DMSO would lead to stable DMSO adducts [6]. The introduction of the *N*-donor leaving groups increased the solubility notably in 1% DMF/PBS from ca. 0.3 mM (similar for all chlorido complexes) to 24.6 mM for **3c**. The osmium chlorido and *N*-donor analogs showed similar solubilities as the respective Ru(II) compounds.

HRMS revealed the counterion-free species [M-NO_3_]^+^ as well as additional MeIm abstraction [M-NO_3_-C_4_H_6_N_2_]^+^ (Figures S11–S20). All complexes showed hygroscopic behavior, confirmed by the obtained oxygen values of the performed elemental analyses.

**Figure 2:**
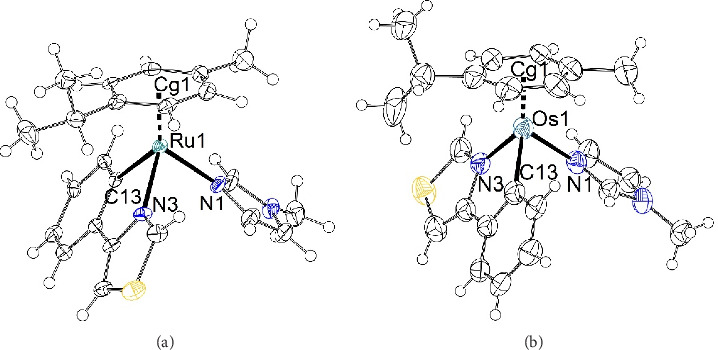
Displacement ellipsoid plot [37] (50% probability) of **3a** (a) one mono-cationic complex [Ru(*p*-cym)(thi)(MeIm)]+ in the asymmetric residue (selected distances (Å) and angles (deg): Ru1–N1 2.098(6), Ru1–N3 2.087(5), Ru1–C13 2.073(7), Ru1⋯Cg1 1.705(3), N3–Ru1–C13 78.2(2)) and **4a** (b) one mono-cationic complex [Os(*p*-cym)(thi)(MeIm)]+ in the asymmetric residue (selected distances (Å) and angles (deg): Os1–N1 2.096(14), Os1–N3 2.071(14), Os1–C13 2.090(18), Os1⋯Cg1 1.713(8), N3–Os1–C13 78.0(6)).

Single crystals of **3a** and **4a** were obtained via slow diffusion or vapor diffusion of Et_2_O in dichloromethane (DCM) complex solutions. Crystal data are displayed in the SI (Table S1). Compound **3a** crystallized as the hemihydrate in the noncentrosymmetric space group *P*2_1_ (Figure 2(a); Figure S21, left).

In both mono-cationic complexes in the asymmetric residue, the central ruthenium(II) cations are coordinated in a piano-stool configuration with an η^6^-*p*-cym, MeIm, and 4-phenylthiazole ligand, each leading to a pseudo-octahedral coordination. If the Ru(II) cations are perceived as stereogenic centers inside their pseudo-octahedral coordination sphere, the two symmetry-independent complexes show the same configuration, leaving only one configuration in the crystal due to symmetry reasons. However, there is a slight shift of about 30° in the *p*-cym coordination (Figure S22).

The nitrate counterion is well-ordered and bridged via a hydrogen bond donated by the co-crystallized water molecule. There are several other nonclassic hydrogen bonds from the complexes' ligands surrounding the nitrate counterion, with the closest being between a nitrate oxygen and an imidazole C-H at around 2.27 Å.

Compound **4a** crystallized as the hemihydrate in the noncentrosymmetric space group *Pc* (Figure 2(b); Figure S21, right). Interestingly, **4a** features four symmetry-independent complexes in the asymmetric residue. This is partially due to the fact that osmium complexes show disordering of the coordinated ligand **a**, which is known for this motif (refcode NOSSAR [38], TODJAA [39]) as the 5-membered thiazole and 6-membered phenyl ring have similar space requirements. The coordination sphere around the four osmium(II) cations is rather similar throughout all the symmetry-independent complexes and also comparable to the coordination observed in **3a**. The nitrate counterion is well-ordered and similar to the observation in compound **3a**. Even if neglecting the disordering of the phenylthiazole ligands, not all complexes exhibit the same stereochemistry in contrast to **3a** due to the glide plane present in space group *Pc*. This is a rather interesting observation, as the complexes in **3a** and **4a** are similar in space requirement and one could expect them to be isomorphous. A comparison of the simulated powder patterns shows no similarities (Figure S23).

### 2.2. In Vitro Antiproliferative Activity in 2D Models

The antiproliferative activity of the ruthenacycles (**3a–e**) and the osmacycles (**4a–e**) was evaluated in human lung adenocarcinoma (A549), colon adenocarcinoma (SW480), and ovarian teratocarcinoma (CH1/PA-1) cells by the colorimetric MTT assay. Due to the substitution of the labile chlorido ligand with MeIm and the concomitant increase in compound stability, the complexes are expected to enter the cells chemically unaltered.

Most of the metallacycles demonstrated pronounced cytotoxic effects with IC_50_ values in the low micromolar range (Table 1, Figures S24 and S25). Overall, the complexes exhibit strongest activity in the broadly chemosensitive CH1/PA-1 cell line, while displaying the lowest activity in the multi–drug-resistant cell line A549. This aligns with the behavior of the recently published parental chlorido complexes (**1a–e** and **2a–e**) [5]. Notably, ruthenacycles, especially **3d** (but with the exception of **3c**), feature lower IC_50_ values than their chlorido counterparts, whereas the antiproliferative activity of osmacycles **4b** and **4e** was slightly reduced. In contrast to the parental chlorido compounds, cytotoxicities of the osmacycles (**4a–e**) were nearly identical to those of their Ru analogs (**3a–e**). Another remarkable observation was that IC_50_ values of the mesyl-bearing compounds **3c** and **4c** massively increased in contrast to **2c**. Two explanations can be proposed for this behavior. Firstly, the complexes **3c** and **4c** might be too inert to interact with the cells due to the stabilizing effect of the electron-withdrawing mesyl group; or secondly, the cellular accumulation of the organometallic compound might be insufficient. Therefore, the aqueous stability and cellular accumulation were studied to clarify the observed inactivity of **3c** and **4c**.

### 2.3. Stability and Reactivity in Aqueous Solution

**Figure 3:**
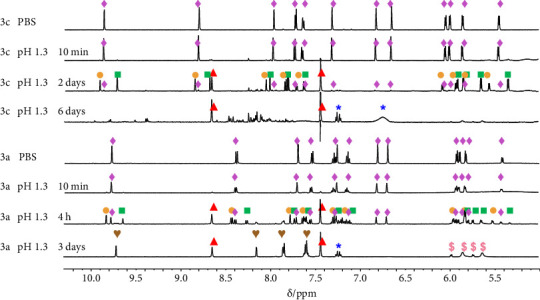
^1^H-NMR spectra of **3a** and **3c** in aqueous medium at pH 7.4 (PBS) and in pH = 1.3 acidic solution followed in time. Symbols: original complex **3a** or **3c** (♦), aqua complex [Ru(*p*-cym) (N,C) (H_2_O)]^+^ (●), chlorido complex [Ru(*p*-cym) (N,C) (Cl)] (■), free HMeIm (▲), free 4-phenylthiazol ligand (♥), free *p*-cymene (^∗^), [Ru(*p*-cym) (Z)]^(2+n)^ ($, Z = H_2_O or Cl^–^). {*c*_complex_ = 1 mM; 10% (v/v) D_2_O/water; 25°C}.

The observed differences in IC_50_ values might be related to the varying stability of the complexes. Hence, the integrity of complexes **3a** and **3c** in aqueous medium was studied with regard to thermodynamic and kinetic aspects in detail by ^1^H-NMR and UV–vis spectroscopy at 25°C. The two complexes showed no decomposition within 6 days at pH 7.4 in aqueous solutions (phosphate-buffered saline, PBS, 1 mM complex concentration) according to the ^1^H-NMR spectra shown in Figure 3. The same behavior was observed in UV–vis experiments conducted at approximately one order of magnitude lower complex concentrations in PBS. The complexes were not sensitive to the chloride or hydroxide ion content of the medium: 3 M KCl could not replace coordinated MeIm at pH 7.4, and also, no spectral change could be observed at pH = 10.0 (NaOH) within 2 days in UV–vis experiments.

Studies on the interaction of amino acids were conducted to elucidate the behavior and potential binding partners of synthesized metallacycles within intricate biological systems. We expect no differences for the affinity toward amino acids compared to the chlorido complexes, but the kinetics are different due to the presence of the MeIm group. Protected amino acids were used to more accurately mimick protein interactions and to circumvent the improbable bidentate or even tridentate chelation that might arise with unprotected amino acids. Therefore, compounds **3a** and **3c** were subjected to incubation with an equimolar mixture (1:1:1) of Ac-L-Met-OMe, Ac-L-His-OMe, and Ac-L-Cys-OMe for 24 h at room temperature. The mixtures were analyzed using ^1^H-NMR (1 mM; 10% dimethylformamide (DMF) in deuterated water (D_2_O)) and HRMS (5 μM; 1% DMF in 400 μM NH_4_OAc solution). Additionally, the ruthenacycles were individually incubated with equimolar amounts of each amino acid, and the resulting interactions were analyzed using the aforementioned ^1^H-NMR method. As expected, no adduct formation was observed and the original complex remained intact over the time frame of the experiments.

From a biological point of view, it may seem unnecessary to study the complexes at strongly acidic pH for several days; however, these experiments were vital to understand the activation processes under slightly acidic (pH∼6), and physiologically more relevant, medium. As the NMR spectra in Figure 3 show, the original complexes are predominant in freshly prepared acidic solutions (pH = 1.0 or 1.3; HCl). However, decomposition of the two complexes **3a** and **3c** occurs with time and in a different manner. The nonsubstituted 4-phenylthiazole complex **3a** tends to hydrolyze into aqua and chlorido complexes ([Ru(*p*-cym)(*N,C*)(H_2_O)]^+^, [Ru*(p*-cym)(*N,C*)(Cl)]) and free HMeIm (protonated MeIm), as observed in the 4-h spectrum (Figure 3). These Ru(*p*-cym) species are not stable, and the bidentate ligand dissociates slowly, resulting in the formation of [Ru(*p*-cym)(*Z*)_3_]^(0/+)^ (*Z* = Cl^−^ or H_2_O) and 4-phenylthiazol as main decomposition products. Furthermore, in the case of **3c**, the MeIm ligand dissociates at pH 1.3. However, the Ru(*p*-cym) bond seems to be destabilized in the resulting aqua and/or chlorido complexes. Consequently, after 6 days the sample contained free *p*-cym, HMeIm, and several minor species in solution, but no liberation of 4-(4-(methylsulfonyl)phenyl)thiazole occurred. UV–vis studies confirmed the above observations (Figure S26).

**Figure 4:**
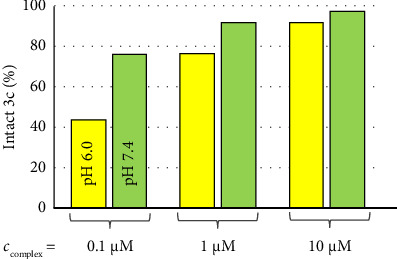
Column diagram showing the nondissociated fraction of **3c** at pH 6.0 (yellow bars) and pH 7.4 (green bars) and at various complex concentrations {*I* = 0.1 M KCl; 25°C}.

In the case of **3c**, a conditional stability constant log*K′* = 8.31 ± 0.03 (*I* = 0.1 M KCl) was computed characterizing the stability of the Ru(*p*-cym)-MeIm bond by considering the protonation of MeIm (p*K*_a_ = 7.13, *I* = 0.1 M KCl) [36]. Calculation was done with HypSpec software based on the pH-dependent UV–vis spectra recorded after 3 days (Figures S27, S28) [40]. With the help of the computed constant, it was possible to model the speciation at various concentrations. Dissociation of the 4-(4-(methylsulfonyl)phenyl)thiazole ligand was not considered, as there was no sign of this process at 100 μM complex concentration.  Figure 4 demonstrates that **3c** is stable at pH 7.4 at 1 μM and 10 μM complex concentrations; even on the submicromolar (0.1 μM) level, the intact complex predominates in solution (78%). At pH 6.0, a condition more characteristic of solid tumor tissues, the MeIm ligand tends to dissociate to a greater extent and only 44% and 76% of the original complex is found in solution at 0.1 and 1 μM concentrations, respectively. A more detailed distribution diagram is provided in Figure S29.

### 2.4. Cellular Accumulation

Another approach to explain the remarkable differences in antiproliferative activity is the determination of the cellular accumulation of the complexes, which was performed in SW480 cells. Additionally, the lipophilicity of the free ligands, serving as a proxy measure for the corresponding complexes, was calculated using Molinspiration and the respective solubilities in 1% DMF/PBS are provided (Table 2). As reported in recent literature, a variation of metal centers showed no relevant impact on the overall lipophilicity of the complexes, which is mainly driven by the ligand sphere [41]. As the coordinated *p*-cym and leaving groups are present in all synthesized complexes, we focused on the differently substituted 4-phenylthiazoles for estimated changes in lipophilicity. Overall, lipophilicity and cellular accumulation of the metallacycles align fairly well (Figure S30). **3b** and **3d** exhibit the highest cell uptake, followed by **3a** and **3e** successively. In contrast, **3c** showed very poor cellular accumulation, which in addition to its stability explains the compound's poor antiproliferative activity in vitro.

### 2.5. Cytotoxicity in Multicellular Tumor Spheroids

Multicellular tumor spheroids provide an improved model to determine the applicability of novel metallodrugs. The colorectal cancer cell line HCT116 was chosen to compare the antiproliferative activity of selected compounds in 2D and 3D cell culture models. Both models were investigated by the fluorimetric resazurin assay after 96-h exposure. IC_50_ values and their 3D/2D quotients (Table 3) indicate that activity is hardly compromised in multicellular spheroids throughout.

A comparison of the parent chlorido complexes **1a** and **1c** suggests that mesylation (**1c**) increases cytotoxic activity by 2.8 times in the 2D setting, while a 3.5-fold enhancement is observed in 3D models. On the other hand, this effect is inverted within the series of MeIm complexes **3a**, **3c**, **4a**, and **4c**. **3a** and **4a** are at least 20-fold more active than their mesylated counterparts **3c** and **4c**, irrespective of the dimensionality of the cell culture model. This is in good agreement with the results of the MTT assay and can be explained by the improved stability of the M-MeIm bond and the limited cellular accumulation of **3c** and **4c**.

### 2.6. Determination of Cellular ROS Levels

After assessing the 2D and 3D antiproliferative activity of the compounds, their capacity of inducing intracellular ROS was studied. For this approach, SW480 cells were subjected to the 2′,7′-dichlorofluorescin-diacetate (DCFH-DA) assay.  Figure S31 shows the time and concentration dependencies of ROS formation by the respective compounds. For all of them, ROS formation to levels higher than in the negative control was hardly noticed, whereas the positive control elevated ROS levels up to more than 3-fold. Nevertheless, some differences between the compounds can be observed. While the parent chlorido complexes **1a** and **1c**, as well as the Ru-MeIm complexes **3a** and **3c**, constantly decrease ROS levels with increasing concentrations, treatment with the Os-MeIm analogs **4a** and **4c** initially lowers ROS levels (in a concentration-dependent manner) which are then restored to nearly normal within 2 hours.

### 2.7. G-Quadruplex Interaction

In a recent work, we demonstrated that the chlorido complexes **1a**, **1c**, and **1d** stabilize DNA G-quadruplex (G4) structures [5]. G4s are DNA secondary motifs that play crucial roles in cancer development [42]. To evaluate the DNA-binding properties of the corresponding MeIm complexes (**3a**, **3c**, and **3d**), we performed fluorescence resonance energy transfer (FRET) melting assays. The complexes were incubated with different G4 forming sequences: one derived from human telomere-forming (h-Telo) and four corresponding to promoter regions of oncogenes, namely, bcl2, hTERT, Kit1, and Kit2. For comparison, a self-complementary sequence called dsDNA was used to represent B-DNA (Table S2). No stabilization of the oligonucleotides, either G4s or B-DNA, was observed after incubation with compounds **3a**, **3c**, and **3d** (Figure S32). This suggests that, while the chlorido complexes can coordinate DNA bases after activation-by-aquation [5], the MeIm moiety of the compounds presented here precludes any interaction with DNA in our experimental conditions.

### 2.8. In Vivo Studies

**Figure 5:**
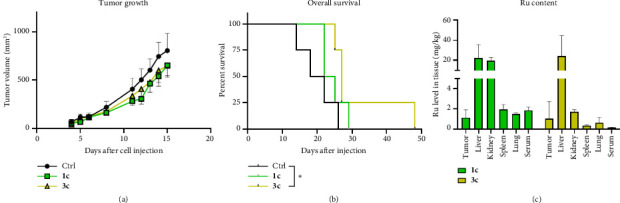
Anticancer activity of **1c** and **3c** in vivo. CT26-bearing BALB/c mice were treated i.p. with 50 mg/kg for five consecutive days for 2 weeks. (a) Impact on tumor growth; data are presented as mean ± SEM. Statistical significance was tested by one-way ANOVA and Tukey's multiple comparison test revealing no statistical significance. On Day 15, most of the animals were sacrificed and tissues collected for further analyses. Of each group, 4 animals were kept to assess the impact on overall survival, which is shown in (b). Statistical significance was tested by log-rank test and Mantel–Cox post-test, ^∗^*p* < 0.01. (c) The ruthenium levels measured by ICP-MS in the collected tissue samples of the mice, which were sacrificed 2 h after the last treatment.

Finally, we were interested in the anticancer activity of selected complexes of our panel in an in vivo mouse setting. Before injecting the compounds in tumor-bearing mice, toxicity experiments were performed. Therefore, Balb/c mice were treated intraperitoneally (i.p.) with 50 mg/kg of complexes **1a, 1c, 3a**, and **3c** for three consecutive days. Due to solubility limitations, the complexes had to be dissolved in 10% DMSO in 0.9% NaCl. Strikingly, the complexes bearing unsubstituted 4-phenylthiazole **1a** and **3a** were highly toxic. Subsequently, for these complexes, finally a dose of only 5 mg/kg was determined as maximal tolerable dose (MTD) for repeated applications. Thus, for the anticancer activity experiments, only **1c** and **3c** were chosen, where CT26 tumor-bearing Balb/c mice were treated 5 times a week for two weeks and the impact on tumor volume monitored by regular caliper measurements. As shown in Figure 5(a), both drugs had a slight anticancer activity, which, however, did not reach statistical significance. On the last day of therapy, most animals were sacrificed and tissues as well as tumors collected. Only four mice (per group) were kept to get information on the impact of the drugs on the overall survival. Here, **3c** resulted in a significantly prolonged overall survival, with one animal experiencing > 200% improved survival (48 days compared to a mean of 20 days in the solvent-treated control animals) (Figure 5(b)).

Tumor-specific organs and blood serum, harvested from the other animals 2 h after the last injection (Figure 5(c)), were measured for the average ruthenium content by inductively coupled plasma mass spectrometry (ICP-MS) of tissue homogenates. Both drugs resulted in similar ruthenium levels in most tissues with ∼1 mg/kg ruthenium in the tumor and ∼20 mg/kg liver. However, striking differences were observed in the kidney, where complex **1c** treatment led to 10-fold higher ruthenium levels compared to **3c**. The presence of high drug levels in organs, which are well blood-supplied and responsible for metabolization and excretion, is very typical for small molecules and thus not unexpected. However, the difference in the kidney levels is interesting, as it might point out a difference in the route of excretion between **1c** and **3c** and, consequently, might explain why **3c** had better long-term therapeutic activity compared to **1c**.

Overall, these collected in vivo results are highly unexpected, as **3c** was the compound with the least activity in the in vitro experiments. Considering that in the case of **1a** and **3a**, the MTD was 10-fold lower compared to **1c** and **3c**, this indicates that the in vitro viability results of the a-series complexes are based more on a general, rather unspecific cell toxicity than on cancer-specific effects.

## 3. Conclusion

In this contribution, ten 4-phenylthiazole-based, MeIm-bearing ruthena(II)- and osma(II)cycles were synthesized. The complexes were characterized by standard analytical techniques, and their interaction with biomolecules was investigated. These studies showed no relevant interaction with selected amino acids or DNA fragments, confirming the increased stability of the MeIm complexes compared to their parental chlorido analogs. Furthermore, the IC_50_ values of all metallacycles were determined in three human cancer cell lines: A549, SW480, CH1/PA-1, and some in HCT116. All complexes proved to be highly active, with IC_50_ values in the low micromolar range, except mesyl-bearing **3c** and **4c**, which showed barely any cytotoxic effect in the tested concentration range. The IC_50_ values of selected compounds in HCT116 multicellular tumor spheroids (3D) were also measured and found to be nearly equivalent to the 2D values. Moreover, the complexes showed no relevant effects on ROS levels, only a slight initial decrease was observed. Stability studies in aqueous solution confirmed the remarkable robustness of MeIm-containing complexes at pH 7.4. Our findings at lower pH values revealed that **3c** disintegrates rather slowly and potentially becomes activated at low concentrations under slightly acidic, tumor-relevant conditions. The inactivity of **3c** in 2D and 3D in vitro settings can be explained by the poor cellular accumulation. Toxicity tests in mice showed that unsubstituted complexes **1a** and **3a** feature very low MTDs, while mesyl-bearing **1c** and **3c** proved to be tolerated at high concentrations of at least 50 mg/kg. The in vivo investigations showed no significant anticancer effect of **1c** and **3c**. However, mice treated with **3c** exhibited the longest overall survival, with one animal showing a survival increase of over 200%, despite the compound's poor in vitro cytotoxicity. Organ distribution revealed markedly different patterns for **1c** and **3c**. Renal Ru levels for **1c** were tenfold higher than those for **3c**, suggesting faster excretion of **1c** and potentially accounting for the extended overall survival observed in mice treated with **3c**, likely due to its extended therapeutic effect.

### 3.1. Experimental Part

#### 3.1.1. Materials and Methods

Chlorido complexes (**1a–e**, **2a–e**) were synthesized as described elsewhere [5]. Single crystals of **3a** and **4a** were obtained via vapor diffusion of Et_2_O into a DCM solution.

All solvents were of analytical grade and used without further purification. NaCl, KCl, 36%(w/w) HCl, KOH, NaOH, NaH_2_PO_4_, Na_2_HPO_4_, and KH_2_PO_4_ and D_2_O were purchased from Sigma-Aldrich in puriss quality. 1-Methylimidazole (99%, Sigma-Aldrich), silver nitrate (99.5%, Acros Fisher), PBS (pH 7.4, 10x, Gibco), Ac-L-Met-OMe (97%, Ambeed), Ac-L-His-OMe (95%, Ambeed), and Ac-L-Cys-OMe (97%, Ambeed) were used without further purification. Ultrapure Milli-Q water was used for sample preparation.

Purification via flash column chromatography was conducted with a Biotage Isolera system and silica gel (VWR, mesh 40–63 μm). ^1^H-, ^13^C-, and 2D-NMR spectra of the complexes were recorded on a Bruker FT-NMR spectrometer Avance III™ HD 700.40 MHz or on a Bruker FT-NMR spectrometer Avance III™ 600.25 MHz. ^1^H-NMR spectra of the ligands and amino acid interaction studies were recorded on a Bruker FT-NMR spectrometer AV NEO 500.10 MHz in CDCl_3_ or D_2_O and referenced to the residual solvent signals. High-resolution ESI mass spectra of the metallacycles were recorded at the Mass Spectrometry Center of the University of Vienna (Faculty of Chemistry) on a Bruker maXis ESI-Qq-TOF mass spectrometer. X-ray intensity data for **3a** were collected at 100(2) K with a STOE Stadivari diffractometer (STOE & CIE GmbH, Germany) equipped with a Dectris EIGER2R 500K area detector (Dectris Ltd., Switzerland), an AXO A-MiXS Mo microsource (*λ* = 0.71073 Å), and an Oxford Cryostream 800 (Oxfordshire, UK). Data were integrated with X-Area(Stoe & Cie, X-Area, 2002, Darmstadt, Germany) and corrected for absorption by the multiscan method with LANA [43]. X-ray intensity data for **4a** were collected at 100(2) K on a Bruker D8 goniometer (Bruker AXS Inc., Madison, Wisconsin, USA) equipped with a Dectris EIGER2R 500K area detector, an Incoatec IµS Diamond Cu microsource (*λ* = 1.54178 Å), and an Oxford Cryostream 800. Data were integrated using the XDS program suite [44] and corrected for absorption by the multiscan method with SADABS-2016/2 [45]. The structures were solved by intrinsic phasing [46] and refined by full matrix least squares procedures based on *F*^2^ as implemented in SHELXL-19 [47]. Hydrogens were treated as riding with C-H = 0.98 Å for CH_3_, C-H = 0.95 Å for aromatic H, and C-H = 1.00 Å for alkyl H. The water hydrogens were placed at calculated positions pointing toward their hydrogen bond acceptor with a bond distance of O-H = 0.85 Å without being refined. The hydrogens were assigned isotropic displacement parameters constrained to *U*_iso_(H) = 1.5 *U*_eq_(C,O) for methyl groups and water hydrogens or *U*_iso_(H) = 1.2 *U*_*eq*_(C) of their parent atoms otherwise. Elemental analyses were performed by the Microanalytical Laboratory of the University of Vienna with a Eurovector EA 3000(2009) equipped with a high-temperature pyrolysis furnace (HT, Hekatech, Germany, 2009). Elemental analysis samples were weighed on a Sartorius SEC 2 ultra-micro balance with ±0.1 μg resolution. Sample weights of 1–3 mg were used. For calibration, two NIST-certified reference materials were used: sulfanilamide (C_6_H_8_N_2_O_2_S) and BBOT (2, 5-bis-(5-tert-butyl-2-benzoxazol-2-yl)-thiophenone, C_26_H_26_N_2_O_2_S). The limit of quantification (LOQ) was 0.05 w-% for C, H, N, and 0.02 w-% for S. The presented values are the average of determinations in triplicate. UV–vis data were recorded on a PerkinElmer Lambda 650 UV–vis Spectrophotometer with a Peltier element for temperature control. FRET experiments were performed at the AteN Center–Università di Palermo using an Applied Biosystems QuantStudio 6 PCR cycler.

### 3.2. General Procedure

The appropriate Ru(II) or Os(II) chlorido complex (1.0 eq.) and 1-methylimidazole (1.0 eq.) were dissolved in MeOH. After the addition of AgNO_3_ (1.5 eq.), the mixture was stirred for 2 h at rt under light exclusion. Subsequently, the solvent was removed under reduced pressure and the crude product was purified via column chromatography on silica (0%–10% MeOH in DCM) to afford the desired metallacycles in elemental analysis purity after drying for 2 days at 50°C in vacuo.

#### 3.2.1. [((3-κN)-1-Methylimidazole)(4-phenylthiazolato-κN,κC2′)(η^6^-*p*-cymene)ruthenium(II)] Nitrate (**3a**)

The reaction was performed according to the general procedure, using the chlorido complex **1a** (200 mg, 464 μmol, 1.0 eq.), 1-methylimidazole (39 mg, 464 μmol, 1.0 eq.), and AgNO_3_ (118 mg, 696 μmol, 1.5 eq.) in MeOH (20 mL). Purification: 10 g SiO_2_, 5% MeOH in DCM; Yield: 228 mg, 91%. ESI-HR-MS^+^*m/z* Found (Calculated): [M–NO_3_]^+^ 478.0894 (478.0891). Elemental analysis found (calculated) for C_23_H_26_N_4_O_3_RuS*·*0.60 H_2_O: C 49.82 (50.19), H 4.75 (4.98), N 9.93 (10.18), S 5.62 (5.83), O 10.28 (10.46). ^1^H-NMR (600.25 MHz, CDCl_3_): *δ* = 10.37 (d, ^4^*J*_*H*,*H*_ = 2 Hz, 1H, ArH_Th-2_), 8.47 (d, ^4^*J*_*H*,*H*_ = 1 Hz, 1H, ArH_MeIm-2_), 8.20 (dd, ^3^*J*_*H*,*H*_ = 8 Hz, ^4^*J*_*H*,*H*_ = 1 Hz, 1H, ArH_Ph-3_), 7.39 (dd, ^3^*J*_*H*,*H*_ = 8 Hz, ^4^*J*_*H*,*H*_ = 1 Hz, 1H, ArH_Ph-6_), 7.24–7.19 (m, 2H, ArH_Th-4_, ArH_Ph-4_), 7.08 (ddd, ^3^*J*_*H*,*H*_ = 7 Hz, ^3^*J*_*H*,*H*_ = 7 Hz, ^4^*J*_*H*,*H*_ = 1 Hz, 1H, ArH_Ph-5_), 6.49–6.46 (m, 1H, ArH_MeIm-5_), 6.32–6.28 (m, 1H, ArH_MeIm-4_), 5.98–5.91 (m, 2H, ArH_Cym-h_, ArH_Cym-g_), 5.65 (d, ^3^*J*_*H*,*H*_ = 6 Hz, 1H, ArH_Cym-f_), 5.54 (d, ^3^*J*_*H*,*H*_ = 6 Hz, 1H, ArH_Cym-e_), 3.62 (s, 3H, CH_3 MeIm_), 2.05 (hept, ^3^*J*_*H*,*H*_ = 7 Hz, 1H, CH_Cym-c_), 1.61 (s, 3H, CH_3 Cym-d_), 0.90 (d, ^3^*J*_*H*,*H*_ = 7 Hz, 3H, CH_3 Cym-b_), 0.74 (d, ^3^*J*_*H*,*H*_ = 7 Hz, 3H, CH_3 Cym-a_) ppm. ^13^C-NMR (150.93 MHz, CDCl_3_): *δ* = 173.9 (C_Ph-2_), 162.6 (C_Th-5_), 157.9 (C_Th-2_), 142.5 (C_MeIm-2_), 139.5 (C_Ph-3_), 139.1 (C_Ph-1_), 130.9 (C_MeIm-4_), 128.8 (C_Ph-4_), 123.8 (C_Ph-5_), 122.6 (C_Ph-6_), 120.5 (C_MeIm-5_), 108.4 (C_Th-4_), 100.1 (C_Cym-1_), 99.7 (C_Cym-6_), 90.7 (C_Cym-g_), 89.8 (C_Cym-f_), 89.4 (C_Cym-h_), 84.6 (C_Cym-e_), 34.6 (C_MeIm-CH3_), 31.0 (C_Cym-c_), 22.5 (C_Cym-b_), 22.1 (C_Cym-a_), 18.2 (C_Cym-d_) ppm.

#### 3.2.2. [((3-κN)-1-Methylimidazole)(4-(4-fluorophenyl)thiazolato-κN,κC2′)(η^6^-*p*-cymene)ruthenium(II)] Nitrate (**3b**)

The reaction was performed according to the general procedure, using the chlorido complex **1b** (243 mg, 541 μmol, 1.0 eq.), 1-methylimidazole (44 mg, 541 μmol, 1.0 eq.), and AgNO_3_ (138 mg, 812 μmol, 1.5 eq.) in MeOH (25 mL). Purification: 10 g SiO_2_, 4% MeOH in DCM; Yield: 206 mg, 68%. ESI-HR-MS^+^ m/z Found (Calculated): [M–NO_3_]^+^ 496.0799 (496.0797). Elemental analysis found (calculated) for C_23_H_25_FN_4_O_3_RuS*·*0.50 H_2_O: C 48.68 (48.75), H 4.51 (4.63), N 9.91 (9.89), S 5.59 (5.66), O 9.63 (9.88). ^1^H-NMR (700.40 MHz, CDCl_3_): *δ* = 10.37 (d, ^4^*J*_*H*,*H*_ = 2 Hz, 1H, ArH_Th-2_), 8.47 (s, 1H, ArH_MeIm-2_), 7.89 (dd, ^3^*J*_*H*,*F*_ = 9 Hz, ^4^*J*_*H*,*H*_ = 3 Hz, 1H, ArH_Ph-3_), 7.37 (dd, ^3^*J*_*H*,*H*_ = 8 Hz, ^4^*J*_*H*,*F*_ = 5 Hz, 1H, ArH_Ph-6_), 7.16 (d, ^4^*J*_*H*,*H*_ = 2 Hz, 1H, ArH_Th-4_), 6.78 (ddd, ^3^*J*_*H*,*F*_ = 9 Hz, ^3^*J*_*H*,*F*_ = 9 Hz, ^4^*J*_*H*,*H*_ = 3 Hz, 1H, ArH_Ph-6_), 6.52–6.51 (m, 1H, ArH_MeIm-5_), 6.33–6.32 (m, 1H, ArH_MeIm-4_), 5.99–5.95 (m, 2H, ArH_Cym-h_, ArH_Cym-g_), 5.64 (d, ^3^*J*_*H*,*H*_ = 6 Hz, 1H, ArH_Cym-f_), 5.51 (d, ^3^*J*_*H*,*H*_ = 6 Hz, 1H, ArH_Cym-e_), 3.63 (s, 3H, CH_3 MeIm_), 2.05 (hept, ^3^*J*_*H*,*H*_ = 7 Hz, 1H, CH_Cym-c_), 1.61 (s, 3H, CH_3 Cym-d_), 0.90 (d, ^3^*J*_*H*,*H*_ = 7 Hz, 3H, CH_3 Cym-b_), 0.74 (d, ^3^*J*_*H*,*H*_ = 7 Hz, 3H, CH_3 Cym-a_) ppm. ^13^C-NMR (176.12 MHz, CDCl_3_): *δ* = 176.7 (d, ^3^*J*_*C*,*F*_ = 3 Hz, C_Ph-2_), 162.1 (d, ^2^*J*_*C*,*F*_ = 252 Hz, C_Ph-4_), 161.6 (C_Th-5_), 158.2 (C_Th-2_), 142.5 (C_MeIm-2_), 135.3 (d, ^4^*J*_*C*,*F*_ = 2 Hz, C_Ph-1_), 130.8 (C_MeIm-4_), 125.2 (d, ^2^*J*_*C*,*F*_ = 17 Hz, C_Ph-3_), 123.7 (d, *J* = 8 Hz, C_Ph-6_), 120.7 (C_MeIm-5_), 110.8 (d, *J* = 23 Hz, C_Ph-5_), 107.9 (C_Th-4_), 100.4 (C_Cym-6_), 100.4 (C_Cym-1_), 90.9 (C_Cym-h_), 89.9 (C_Cym-g_), 89.8 (C_Cym-f_), 84.5 (C_Cym-e_), 34.7 (C_MeIm-CH3_), 31.0 (C_Cym-c_), 22.6 (C_Cym-b_), 22.0 (C_Cym-a_), 18.3 (C_Cym-d_) ppm.

#### 3.2.3. [((3-κN)-1-Methylimidazole)(4-(4-(methylsulfonyl)phenyl)thiazolato-κN,κC2′)(η^6^-*p*-cymene)ruthenium(II)] Nitrate (**3c**)

The reaction was performed according to the general procedure, using the chlorido complex **1c** (300 mg, 589 μmol, 1.0 eq.), 1-methylimidazole (48 mg, 589 μmol, 1.0 eq.), and AgNO_3_ (150 mg, 884 μmol, 1.5 eq.) in MeOH (30 mL). Purification: 10 g SiO_2_, 8% MeOH in DCM; Yield: 266 mg, 73%. ESI-HR-MS^+^ m/z Found (Calculated): [M–NO_3_]^+^ 556.0668 (556.0666). Elemental analysis found (calculated) for C_24_H_28_N_4_O_5_RuS_2_*·*0.75 H_2_O: C 45.28 (45.67), H 4.60 (4.71), N 8.89 (8.88), S 10.06 (10.16), O 14.51 (14.57). ^1^H-NMR (600.25 MHz, CDCl_3_): *δ* = 10.56 (d, ^4^*J*_*H*,*H*_ = 2 Hz, 1H, ArH_Th-2_), 8.74 (d, ^4^*J*_*H*,*H*_ = 2 Hz, 1H, ArH_Ph-3_), 8.45 (s, 1H, ArH_MeIm-2_), 7.63 (dd, ^3^*J*_*H*,*H*_ = 8 Hz, ^4^*J*_*H*,*H*_ = 2 Hz, 1H, ArH_Ph-5_), 7.53 (d, ^3^*J*_*H*,*H*_ = 8 Hz, 1H, ArH_Ph-6_), 7.47 (d, ^4^*J*_*H*,*H*_ = 2 Hz, 1H, ArH_Th-4_), 6.54–6.50 (m, 1H, ArH_MeIm-5_), 6.24 (s, 1H, ArH_MeIm-4_), 6.13–6.03 (m, 2H, ArH_Cym-h_, ArH_Cym-g_), 5.66 (d, ^3^*J*_*H*,*H*_ = 6 Hz, 1H, ArH_Cym-f_), 5.54 (d, ^3^*J*_*H*,*H*_ = 6 Hz, 1H, ArH_Cym-e_), 3.63 (s, 3H, CH_3 MeIm_), 3.19 (s, 3H, CH_3 SO2Me_), 2.07 (hept, ^3^*J*_*H*,*H*_ = 7 Hz, 1H, CH_Cym-c_), 1.64 (s, 3H, CH_3 Cym-d_), 0.89 (d, ^3^*J*_*H*,*H*_ = 7 Hz, 3H, CH_3 Cym-b_), 0.72 (d, ^3^*J*_*H*,*H*_ = 7 Hz, 3H, CH_3 Cym-a_) ppm. ^13^C-NMR (150.93 MHz, CDCl_3_): *δ* = 175.5 (C_Ph-2_), 160.8 (C_Th-5_), 159.3 (C_Th-2_), 144.1 (C_Ph-1_), 142.5 (C_MeIm-2_), 139.3 (C_Ph-4_), 136.9 (C_Ph-3_), 130.9 (C_MeIm-4_), 123.0 (C_Ph-5_), 122.7 (C_Ph-6_), 120.9 (C_MeIm-5_), 112.1 (C_Th-4_), 101.9 (C_Cym-6_), 100.7 (C_Cym-1_), 91.6 (C_Cym-h_), 90.5 (C_Cym-g_), 89.9 (C_Cym-f_), 83.6 (C_Cym-e_), 45.0 (C_SO2Me_), 34.8 (C_MeIm-CH3_), 31.0 (C_Cym-c_), 22.7 (C_Cym-b_), 22.0 (C_Cym-a_), 18.3 (C_Cym-d_) ppm.

#### 3.2.4. [((3-κN)-1-Methylimidazole)(4-(4-methylphenyl)thiazolato-κN,κC2′)(η^6^-*p*-cymene)ruthenium(II)] Nitrate (**3d**)

The reaction was performed according to the general procedure, using the chlorido complex **1d** (200 mg, 449 μmol, 1.0 eq.), 1-methylimidazole (37 mg, 449 μmol, 1.0 eq.), and AgNO_3_ (115 mg, 674 mmol, 1.5 eq.) in MeOH (20 mL). Purification: 10 g SiO_2_, 6% MeOH in DCM; Yield: 178 mg, 71%. ESI-HR-MS^+^ m/z Found (Calculated): [M–NO_3_]^+^ 492.1044 (492.1048). Elemental analysis found (calculated) for C_24_H_28_N_4_O_3_RuS*·*0.40 H_2_O: C 51.21 (51.40), H 5.13 (5.18), N 9.66 (9.99), S 5.48 (5.72), O 9.47 (9.70). ^1^H-NMR (600.25 MHz, CDCl_3_): *δ* = 10.32 (d, ^4^*J*_*H*,*H*_ = 2 Hz, 1H, ArH_Th-2_), 8.48 (s, 1H, ArH_MeIm-2_), 8.01 (s, 1H, ArH_Ph-3_), 7.28 (d, ^3^*J*_*H*,*H*_ = 8 Hz, 1H, ArH_Ph-6_), 7.14 (d, ^4^*J*_*H*,*H*_ = 2 Hz, 1H, ArH_Th-4_), 6.89 (dd, ^3^*J*_*H*,*H*_ = 8 Hz, ^4^*J*_*H*,*H*_ = 2 Hz, 1H, ArH_Ph-5_), 6.49–6.45 (m, 1H, ArH_MeIm-5_), 6.32–6.28 (m, 1H, ArH_MeIm-4_), 5.97 (dd, ^3^*J*_*H*,*H*_ = 6 Hz, ^4^*J*_*H*,*H*_ = 1 Hz, 1H, ArH_Cym-h_), 5.92 (dd, ^3^*J*_*H*,*H*_ = 6 Hz, ^4^*J*_*H*,*H*_ = 1 Hz, 1H, ArH_Cym-g_), 5.63 (dd, ^3^*J*_*H*,*H*_ = 6 Hz, ^4^*J*_*H*,*H*_ = 1 Hz, 1H, ArH_Cym-f_), 5.52 (dd, ^3^*J*_*H*,*H*_ = 6 Hz, ^4^*J*_*H*,*H*_ = 1 Hz, 1H, ArH_Cym-e_), 3.62 (s, 3H, CH_3 MeIm_), 2.45 (s, 3H, CH_3 Ph_), 2.05 (hept, ^3^*J*_*H*,*H*_ = 7 Hz, 1H, CH_Cym-c_), 1.60 (s, 3H, CH_3 Cym-d_), 0.90 (d, ^3^*J*_*H*,*H*_ = 7 Hz, 3H, CH_3 Cym-b_), 0.73 (d, ^3^*J*_*H*,*H*_ = 7 Hz, 3H, CH_3 Cym-a_) ppm. ^13^C-NMR (150.93 MHz, CDCl_3_): *δ* = 173.9 (C_Ph-2_), 162.6 (C_Th-5_), 157.6 (C_Th-2_), 142.6 (C_MeIm-2_), 140.0 (C_Ph-3_), 138.4 (C_Ph-4_), 136.4 (C_Ph-1_), 130.8 (C_MeIm-4_), 124.8 (C_Ph-5_), 122.3 (C_Ph-6_), 120.4 (C_MeIm-5_), 107.4 (C_Th-4_), 99.9 (C_Cym-1_), 99.4 (C_Cym-6_), 90.8 (C_Cym-g_), 89.6 (C_Cym-h_), 89.5 (C_Cym-f_), 84.5 (C_Cym-e_), 34.6 (C_MeIm-CH3_), 31.0 (C_Cym-c_), 22.6 (C_Cym-b_), 22.1 (C_Cym-a_), 22.0 (C_CH3_), 18.2 (C_Cym-d_) ppm.

#### 3.2.5. [((3-κN)-1-Methylimidazole)(4-(4-methoxyphenyl)thiazolato-κN,κC2′)(η^6^-*p*-cymene)ruthenium(II)] Nitrate (**3e**)

The reaction was performed according to the general procedure, using the chlorido complex **1e** (200 mg, 434 μmol, 1.0 eq.), 1-methylimidazole (36 mg, 434 μmol, 1.0 eq.), and AgNO_3_ (111 mg, 651 μmol, 1.5 eq.) in MeOH (20 mL). Purification: 10 g SiO_2_, 5% MeOH in DCM; Yield: 205 mg, 85%. ESI-HR-MS^+^ m/z Found (Calculated): [M–NO_3_]^+^ 508.0998 (508.0997). Elemental analysis found (calculated) for C_24_H_28_N_4_O_4_RuS*·*0.35 H_2_O: C 49.67 (50.05), H 4.95 (5.02), N 9.55 (9.73), S 5.47 (5.57), O 11.68 (12.08). ^1^H-NMR (700.40 MHz, CDCl_3_): *δ* = 10.29 (d, ^3^*J*_*H*,*H*_ = 2 Hz, 1H, ArH_Th-2_), 8.46 (s, 1H, ArH_MeIm-2_), 7.76 (d, ^4^*J*_*H*,*H*_ = 3 Hz, 1H, ArH_Ph-3_), 7.34 (d, ^3^*J*_*H*,*H*_ = 8 Hz, 1H, ArH_Ph-6_), 7.05 (d, ^4^*J*_*H*,*H*_ = 2 Hz, 1H, ArH_Th-4_), 6.64 (dd, ^3^*J*_*H*,*H*_ = 8 Hz, ^4^*J*_*H*,*H*_ = 3 Hz, 1H, ArH_Ph-5_), 6.50–6.47 (m, 1H, ArH_MeIm-5_), 6.38–6.35 (m, 1H, ArH_MeIm-4_), 5.96 (d, ^3^*J*_*H*,*H*_ = 6 Hz, 1H, ArH_Cym-h_), 5.92 (d, ^3^*J*_*H*,*H*_ = 6 Hz, 1H, ArH_Cym-g_), 5.64 (d, ^3^*J*_*H*,*H*_ = 6 Hz, 1H, ArH_Cym-f_), 5.52 (d, ^3^*J*_*H*,*H*_ = 6 Hz, 1H, ArH_Cym-e_), 3.93 (s, 3H, CH_3 OMe_), 3.62 (s, 3H, CH_3 MeIm_), 2.05 (hept, ^3^*J*_*H*,*H*_ = 7 Hz, 1H, CH_Cym-c_), 1.60 (s, 3H, CH_3 Cym-d_), 0.90 (d, ^3^*J*_*H*,*H*_ = 7 Hz, 3H, CH_3 Cym-b_), 0.74 (d, ^3^*J*_*H*,*H*_ = 7 Hz, 3H, CH_3 Cym-a_) ppm. ^13^C-NMR (176.12 MHz, CDCl_3_): *δ* = 175.7 (C_Ph-2_), 162.2 (C_Th-5_), 159.2 (C_Ph-4_), 157.6 (C_Th-2_), 142.5 (C_MeIm-2_), 132.5 (C_Ph-1_), 130.8 (C_MeIm-4_), 125.1 (C_Ph-3_), 123.3 (C_Ph-6_), 120.5 (C_MeIm-5_), 108.1 (C_Ph-5_), 106.3 (C_Th-4_), 100.1 (C_Cym-1_), 99.7 (C_Cym-6_), 90.6 (C_Cym-g_), 89.7 (C_Cym-h_), 89.7 (C_Cym-f_), 84.7 (C_Cym-e_), 55.4 (C_OCH3_), 34.6 (C_MeIm-CH3_), 31.0 (C_Cym-c_), 22.6 (C_Cym-b_), 22.1 (C_Cym-a_), 18.2 (C_Cym-d_) ppm.

#### 3.2.6. [((3-κN)-1-Methylimidazole)(4-phenylthiazolato-κN,κC2′)(η^6^-*p*-cymene)osmium(II)] Nitrate (**4a**)

The reaction was performed according to the general procedure, using the chlorido complex **2a** (200 mg, 385 μmol, 1.0 eq.), 1-methylimidazole (32 mg, 385 μmol, 1.0 eq.), and AgNO_3_ (98 mg, 577 μmol, 1.5 eq.) in MeOH (20 mL). Purification: 10 g SiO_2_, 6% MeOH in DCM; Yield: 223 mg, 92%. ESI-HR-MS^+^ m/z Found (Calculated): [M–NO_3_]^+^ 568.1459 (568.1455). Elemental analysis found (calculated) for C_23_H_26_N_4_O_3_OsS*·*0.50 H_2_O: C 43.01 (43.32), H 3.99 (4.27), N 8.74 (8.78), S 4.93 (5.03), O 8.74 (8.78). ^1^H-NMR (600.25 MHz, CDCl_3_): *δ* = 10.27 (d, ^3^*J*_*H*,*H*_ = 2 Hz, 1H, ArH_Th-2_), 8.55 (d, ^4^*J*_*H*,*H*_ = 2 Hz, 1H, ArH_MeIm-2_), 8.08 (dd, ^3^*J*_*H*,*H*_ = 8 Hz, ^4^*J*_*H*,*H*_ = 1 Hz, 1H, ArH_Ph-3_), 7.43 (dd, ^3^*J*_*H*,*H*_ = 8 Hz, ^4^*J*_*H*,*H*_ = 1 Hz, 1H, ArH_Ph-6_), 7.21 (d, ^3^*J*_*H*,*H*_ = 2 Hz, 1H, ArH_Th-4_), 7.16 (ddd, ^3^*J*_*H*,*H*_ = 7 Hz, ^4^*J*_*H*,*H*_ = 1 Hz, ^4^*J*_*H*,*H*_ = 1 Hz, 1H, ArH_Ph-4_), 7.08 (ddd, ^3^*J*_*H*,*H*_ = 7 Hz, ^4^*J*_*H*,*H*_ = 1 Hz, ^4^*J*_*H*,*H*_ = 1 Hz, 1H, ArH_Ph-5_), 6.42 (m, 1H, ArH_MeIm-5_), 6.33 (m, 1H, ArH_MeIm-4_), 5.98 (dd, ^3^*J*_*H*,*H*_ = 6 Hz, ^4^*J*_*H*,*H*_ = 1 Hz, 1H, ArH_Cym-h_), 5.82 (dd, ^3^*J*_*H*,*H*_ = 6 Hz, ^4^*J*_*H*,*H*_ = 1 Hz, 1H, ArH_Cym-g_), 5.63 (dd, ^3^*J*_*H*,*H*_ = 6 Hz, ^4^*J*_*H*,*H*_ = 1 Hz, 1H, ArH_Cym-f_), 5.56 (dd, ^3^*J*_*H*,*H*_ = 5 Hz, ^4^*J*_*H*,*H*_ = 1 Hz, 1H, ArH_Cym-e_), 3.64 (s, 3H, CH_3 MeIm_), 2.06 (hept, ^3^*J*_*H*,*H*_ = 7 Hz, 1H, CH_Cym-c_), 1.76 (s, 3H, CH_3 Cym-d_), 0.91 (d, ^3^*J*_*H*,*H*_ = 7 Hz, 3H, CH_3 Cym-b_), 0.76 (d, ^3^*J*_*H*,*H*_ = 7 Hz, 3H, CH_3 Cym-a_) ppm. ^13^C-NMR (150.93 MHz, CDCl_3_): *δ* = 165.2 (C_Th-5_), 160.1 (C_Ph-2_), 157.7 (C_Th-2_), 143.2 (C_MeIm-2_), 139.6 (C_Ph-1_), 139.5 (C_Ph-3_), 130.3 (C_MeIm-4_), 129.4 (C_Ph-4_), 123.8 (C_Ph-5_), 122.5 (C_Ph-6_), 120.3 (C_MeIm-5_), 108.4 (C_Th-4_), 92.6 (C_Cym-6_), 90.9 (C_Cym-1_), 80.9 (C_Cym-g_), 80.6 (C_Cym-e_), 80.2 (C_Cym-h_), 74.3 (C_Cym-f_), 34.6 (C_MeIm-CH3_), 31.1 (C_Cym-c_), 22.8 (C_Cym-b_), 22.5(C_Cym-a_), 18.0 (C_Cym-d_) ppm.

#### 3.2.7. [((3-κN)-1-Methylimidazole)(4-(4-fluorophenyl)thiazolato-κN,κC2′)(η^6^-*p*-cymene)osmium(II)] Nitrate (**4b**)

The reaction was performed according to the general procedure, using the chlorido complex **2b** (226 mg, 420 μmol, 1.0 eq.), 1-methylimidazole (34 mg, 420 μmol, 1.0 eq.), and AgNO_3_ (107 mg, 630 μmol, 1.5 eq.) in MeOH (20 mL). Purification: 10 g SiO_2_, 6% MeOH in DCM; Yield: 177 mg, 67%. ESI-HR-MS^+^ m/z Found (Calculated): [M–NO_3_]^+^ 586.1358 (586.1361). Elemental analysis found (calculated) for C_23_H_25_N_4_FO_3_OsS*·*0.75 H_2_O: C 41.70 (41.84), H 3.80 (4.05), N 8.49 (8.49), S 4.62 (4.86), O 8.74 (9.09). ^1^H-NMR (600.25 MHz, CDCl_3_): *δ* = 10.29 (d, ^3^*J*_*H*,*H*_ = 2 Hz, 1H, ArH_Th-2_), 8.56 (s, 1H, ArH_MeIm-2_), 7.75 (dd, ^3^*J*_*H*,*F*_ = 9 Hz, ^4^*J*_*H*,*H*_ = 3 Hz, 1H, ArH_Ph-3_), 7.41 (dd, ^3^*J*_*H*,*H*_ = 8 Hz, ^4^*J*_*H*,*F*_ = 6 Hz, 1H, ArH_Ph-6_), 7.14 (d, ^4^*J*_*H*,*H*_ = 2 Hz, 1H, ArH_Th-4_), 6.77 (ddd, ^3^*J*_*H*,*F*_ = 9 Hz, ^3^*J*_*H*,*F*_ = 9 Hz, ^4^*J*_*H*,*H*_ = 3 Hz, 1H, ArH_Ph-5_), 6.47–6.44 (m, 1H, ArH_MeIm-5_), 6.39–6.34 (m, 1H, ArH_MeIm-4_), 6.00 (d, ^3^*J*_*H*,*H*_ = 6 Hz, 1H, ArH_Cym-h_), 5.85 (d, ^3^*J*_*H*,*H*_ = 6 Hz, 1H, ArH_Cym-g_), 5.61 (d, ^3^*J*_*H*,*H*_ = 6 Hz, 1H, ArH_Cym-f_), 5.56 (d, ^3^*J*_*H*,*H*_ = 6 Hz, 1H, ArH_Cym-e_), 3.65 (s, 3H, CH_3 MeIm_), 2.05 (hept, ^3^*J*_*H*,*H*_ = 7 Hz, 1H, C_H Cym-c_), 1.76 (s, 3H, CH_3 Cym-d_), 0.92 (d, ^3^*J*_*H*,*H*_ = 7 Hz, 3H, CH_3 Cym-b_), 0.76 (d, ^3^*J*_*H*,*H*_ = 7 Hz, 3H, CH_3 Cym-a_) ppm. ^13^C-NMR (150.93 MHz, CDCl_3_): *δ* = 164.1 (C_Th-5_), 163.0 (d, ^1^*J*_*C*,*F*_ = 253 Hz, C_Ph-4_), 162.7 (d, ^3^*J*_*C*,*F*_ = 4 Hz, C_Ph-2_), 158.0 (C_Th-2_), 143.2 (C_MeIm-2_), 135.9 (d, ^4^*J*_*C*,*F*_ = 2 Hz, C_Ph-1_), 130.3 (C_MeIm-4_), 125.3 (d, ^2^*J*_*C*,*F*_ = 17 Hz, C_Ph-3_), 123.8 (d, ^3^*J*_*C*,*F*_ = 9 Hz, C_Ph-6_), 120.4 (C_MeIm-5_), 110.8 (d, ^2^*J*_*H*,*F*_ = 23 Hz, C_Ph-5_), 107.9 (C_Th-4_), 93.2 (C_Cym-6_), 91.3 (C_Cym-1_), 81.1 (C_Cym-g_), 80.8 (C_Cym-h_), 80.6 (C_Cym-e_), 74.3 (C_Cym-f_), 34.7 (C_MeIm-CH3_), 31.2 (C_Cym-c_), 22.9 (C_Cym-b_), 22.5 (C_Cym-a_), 18.0 (C_Cym-d_) ppm.

#### 3.2.8. [((3-κN)-1-Methylimidazole)(4-(4-(methylsulfonyl)phenyl)thiazolato-κN,κC2′)(η^6^-*p*-cymene)osmium(II)] Nitrate (**4c**)

The reaction was performed according to the general procedure, using the chlorido complex **2c** (210 mg, 351 μmol, 1.0 eq.), 1-methylimidazole (29 mg, 351 μmol, 1.0 eq.), and AgNO_3_ (89 mg, 527 μmol, 1.5 eq.) in MeOH (20 mL). Purification: 10 g SiO_2_, 9% MeOH in DCM; Yield: 193 mg, 78%. ESI-HR-MS^+^ m/z Found (Calculated): [M–NO_3_]^+^ 646.1233 (646.1229). Elemental analysis found (calculated) for C_24_H_28_N_4_O_5_OsS_2_· 0.50 H_2_O: C 39.76 (40.01), H 4.03 (4.13), N 7.83 (7.78), S 8.70 (8.90), O 12.49 (12.77). ^1^H-NMR (600.25 MHz, CDCl_3_): *δ* = 10.49 (d, ^4^*J*_*H*,*H*_ = 2 Hz, 1H, ArH_Th-2_), 8.60 (d, ^4^*J*_*H*,*H*_ = 2 Hz, 1H, ArH_Ph-3_), 8.54 (s, 1H, ArH_MeIm-2_), 7.62 (dd, ^3^*J*_*H*,*H*_ = 8 Hz, ^4^*J*_*H*,*H*_ = 2 Hz, 1H, ArH_Ph-5_), 7.57 (d, ^3^*J*_*H*,*H*_ = 8 Hz, 1H, ArH_Ph-6_), 7.46 (d, ^3^*J*_*H*,*H*_ = 2 Hz, 1H, ArH_Th-4_), 6.49–6.45 (m, 1H, ArH_MeIm-5_), 6.30–6.27 (m, 1H, ArH_MeIm-4_), 6.09 (d, ^3^*J*_*H*,*H*_ = 6 Hz, 1H, ArH_Cym-h_), 5.97 (d, ^3^*J*_*H*,*H*_ = 6 Hz, 1H, ArH_Cym-g_), 5.66 (d, ^3^*J*_*H*,*H*_ = 6 Hz, 1H, ArH_Cym-f_), 5.58 (d, ^3^*J*_*H*,*H*_ = 6 Hz, 1H, ArH_Cym-e_), 3.65 (s, 3H, CH_3 MeIm_), 3.17 (s, 3H, CH_3 SO2Me_), 2.06 (hept, ^3^*J*_*H*,*H*_ = 7 Hz, 1H, CH_Cym-c_), 1.77 (s, 3H, CH_3 Cym-d_), 0.90 (d, ^3^*J*_*H*,*H*_ = 7 Hz, 3H, CH_3 Cym-b_), 0.73 (d, ^3^*J*_*H*,*H*_ = 7 Hz, 3H, CH_3 Cym-a_) ppm. ^13^C-NMR (150.93 MHz, CDCl_3_): *δ* = 163.3 (C_Th-5_), 161.2 (C_Ph-2_), 159.1 (C_Th-2_), 144.7 (C_Ph-1_), 143.0 (C_MeIm-2_), 139.9 (C_Ph-4_), 137.1 (C_Ph-3_), 130.4 (C_MeIm-4_), 122.9 (C_Ph-5_), 122.7 (C_Ph-6_), 120.7 (C_MeIm-5_), 112.2 (C_Th-4_), 94.7 (C_Cym-6_), 91.5 (C_Cym-1_), 81.9 (C_Cym-g_), 81.5 (C_Cym-h_), 80.8 (C_Cym-e_), 73.5 (C_Cym-f_), 44.9 (C_SO2CH3_), 34.8 (C_MeIm-CH3_), 31.2 (C_Cym-c_), 23.0 (C_Cym-b_), 22.4 (C_Cym-a_), 17.9 (C_Cym-d_) ppm.

#### 3.2.9. [((3-κN)-1-Methylimidazole)(4-(4-methylphenyl)thiazolato-κN,κC2′)(η^6^-*p*-cymene)osmium(II)] Nitrate (**4d**)

The reaction was performed according to the general procedure, using the chlorido complex **2d** (200 mg, 374 μmol, 1.0 eq.), 1-methylimidazole (31 mg, 385 μmol, 1.0 eq.), and AgNO_3_ (95 mg, 562 μmol, 1.5 eq.) in MeOH (20 mL). Purification: 10 g SiO_2_, 6% MeOH in DCM; Yield: 221 mg, 92%. ESI-HR-MS^+^ m/z Found (Calculated): [M–NO_3_]^+^ 582.1614 (582.1612). Elemental analysis found (calculated) for C_24_H_28_N_4_O_3_OsS*·*0.50 H_2_O: C 44.17 (44.22), H 4.27 (4.48), N 8.53 (8.60), S 4.85 (4.92), O 8.71 (8.59). ^1^H-NMR (600.25 MHz, CDCl_3_): *δ* = 10.23 (d, ^4^*J*_*H*,*H*_ = 2 Hz, 1H, ArH_Th-2_), 8.55 (s, 1H, ArH_MeIm-2_), 7.89 (s, 1H, ArH_Ph-3_), 7.32 (d, ^3^*J*_*H*,*H*_ = 8 Hz, 1H, ArH_Ph-6_), 7.12 (d, ^4^*J*_*H*,*H*_ = 2 Hz, 1H, ArH_Th-4_), 6.89 (d, ^3^*J*_*H*,*H*_ = 8 Hz, 1H, ArH_Ph-5_), 6.43–6.40 (m, 1H, ArH_MeIm-5_), 6.36–6.32 (m, 1H, ArH_MeIm-4_), 6.00 (d, ^3^*J*_*H*,*H*_ = 6 Hz, 1H, ArH_Cym-h_), 5.83 (d, ^3^*J*_*H*,*H*_ = 6 Hz, 1H, ArH_Cym-g_), 5.62 (d, ^3^*J*_*H*,*H*_ = 6 Hz, 1H, ArH_Cym-f_), 5.55 (d, ^3^*J*_*H*,*H*_ = 5 Hz, 1H, ArH_Cym-e_), 3.64 (s, 3H, CH_3 MeIm_), 2.42 (s, 3H, CH_3 Ph_), 2.05 (hept, ^3^*J*_*H*,*H*_ = 7 Hz, 1H, CH_Cym-c_), 1.75 (s, 3H, CH_3 Cym-d_), 0.91 (d, ^3^*J*_*H*,*H*_ = 7 Hz, 3H, CH_3 Cym-b_), 0.75 (d, ^3^*J*_*H*,*H*_ = 7 Hz, 3H, CH_3 Cym-a_) ppm. ^13^C-NMR (150.93 MHz, CDCl_3_): *δ* = 165.2 (C_Th-5_), 160.2 (C_Ph-2_), 157.5 (C_Th-2_), 143.2 (C_MeIm-2_), 140.1 (C_Ph-3_), 139.0 (C_Ph-4_), 137.0 (C_Ph-1_), 130.3 (C_MeIm-4_), 124.8 (C_Ph-5_), 122.2 (C_Ph-6_), 120.2 (C_MeIm-5_), 107.4 (C_Th-4_), 92.3 (C_Cym-6_), 90.8 (C_Cym-1_), 81.0 (C_Cym-g_), 80.4 (m, C_Cym-e_, C_Cym-h_), 74.2 (C_Cym-f_), 34.6 (C_MeIm-CH3_), 31.2 (C_Cym-c_), 22.9 (C_Cym-b_), 22.5 (C_Cym-a_), 21.9 (C_CH3_), 18.0 (C_Cym-d_) ppm.

#### 3.2.10. [((3-κN)-1-Methylimidazole)(4-(4-methoxyphenyl)thiazolato-κN,κC2′)(η^6^-*p*-cymene)osmium(II)] Nitrate (**4e**)

The reaction was performed according to the general procedure, using the chlorido complex **2e** (200 mg, 364 μmol, 1.0 eq.), 1-methylimidazole (30 mg, 364 μmol, 1.0 eq.), and AgNO_3_ (93 mg, 545 μmol, 1.5 eq.) in MeOH (20 mL). Purification: 10 g SiO_2_, 7% MeOH in DCM; Yield: 191 mg, 80%. ESI-HR-MS^+^ m/z Found (Calculated): [M–NO_3_]^+^ 598.1564 (598.1561). Elemental analysis found (calculated) for C_24_H_28_N_4_O_4_OsS*·*0.60 H_2_O: C 42.67 (43.05), H 4.17 (4.40), N 8.31 (8.37), S 4.79 (4.79), O 10.79 (10.99). ^1^H-NMR (600.25 MHz, CDCl_3_): *δ* = 10.22 (d, ^4^*J*_*H*,*H*_ = 2 Hz, 1H, ArH_Th-2_), 8.55 (s, 1H, ArH_MeIm-2_), 7.63 (d, ^4^*J*_*H*,*H*_ = 3 Hz, 1H, ArH_Ph-3_), 7.39 (d, ^3^*J*_*H*,*H*_ = 8 Hz, 1H, ArH_Ph-6_), 7.04 (d, ^4^*J*_*H*,*H*_ = 2 Hz, 1H, ArH_Th-4_), 6.64 (dd, ^3^*J*_*H*,*H*_ = 8 Hz, ^4^*J*_*H*,*H*_ = 3 Hz, 1H, ArH_Ph-5_), 6.44–6.42 (m, 1H, ArH_MeIm-5_), 6.42–6.39 (m, 1H, ArH_MeIm-4_), 5.98 (d, ^3^*J*_*H*,*H*_ = 6 Hz, 1H, ArH_Cym-h_), 5.81 (d, ^3^*J*_*H*,*H*_ = 6 Hz, 1H, ArH_Cym-g_), 5.62 (d, ^3^*J*_*H*,*H*_ = 6 Hz, 1H, ArH_Cym-f_), 5.55 (d, ^3^*J*_*H*,*H*_ = 6 Hz, 1H, ArH_Cym-e_), 3.91 (s, 3H, CH_3 MeIm_), 3.64 (s, 3H, CH_3 OMe_), 2.06 (hept, ^3^*J*_*H*,*H*_ = 7 Hz, 1H, CH_Cym-c_), 1.75 (s, 3H, CH_3 Cym-d_), 0.92 (d, ^3^*J*_*H*,*H*_ = 7 Hz, 3H, CH_3 Cym-b_), 0.76 (d, ^3^*J*_*H*,*H*_ = 7 Hz, 3H, CH_3 Cym-a_) ppm. ^13^C-NMR (150.93 MHz, CDCl_3_): *δ* = 164.8 (C_Th-5_), 161.8 (C_Ph-2_), 160.0 (C_Ph-4_), 157.5 (C_Th-2_), 143.2 (C_MeIm-2_), 133.1 (C_Ph-1_), 130.2 (C_MeIm-4_), 125.1 (C_Ph-3_), 123.4 (C_Ph-6_), 120.3 (C_MeIm-5_), 108.2 (C_Ph-5_), 106.3 (C_Th-4_), 92.5 (C_Cym-6_), 91.0 (C_Cym-1_), 80.8 (C_Cym-g_), 80.5 (m, C_Cym-e_, C_Cym-h_) 74.5 (C_Cym-f_), 55.3 (C_OCH3_), 34.6 (C_MeIm-CH3_), 31.1 (C_Cym-c_), 22.9 (C_Cym-b_), 22.5 (C_Cym-a_), 18.0 (C_Cym-d_) ppm.

### 3.3. Cell Culture

CH1/PA-1 ovarian teratocarcinoma cells (CH1, RRID: CVCL_4992, provided by L. R. Kelland, CRC Center for Cancer Therapeutics, Institute of Cancer Research, Sutton, UK; confirmed by STR profiling as PA-1 ovarian teratocarcinoma cells at Multiplexion, Heidelberg, Germany), SW480 colon adenocarcinoma (RRID: CVCL_0546), and A549 lung adenocarcinoma cells (NCI-H2618, RRID: CVCL_A549; both cell lines were obtained from the American Type Culture Collection (ATCC), Manassas, VA, USA) were grown as adherent cultures in 75-cm^2^ culture flasks (Starlab, Hamburg, Germany) by using minimal essential medium (MEM) supplemented with 1 mM sodium pyruvate, 4 mM L-glutamine, 1% (v/v) nonessential amino acids from 100-fold stock (all purchased from Sigma-Aldrich), and 10% heat-inactivated fetal bovine serum (Serana, Pessin, Germany). HCT116 colorectal carcinoma cells (RRID:CVCL_0291 from ATCC) were cultivated in McCoy's 5A medium, supplemented with 10% heat-inactivated fetal bovine serum and 4 mM L-glutamine.

Cells were maintained under standard culture conditions at 37°C in a humidified atmosphere with 5% CO_2_.

### 3.4. MTT Assay

The 3-(4,5-dimethylthiazol-2-yl)-2,5-diphenyl-2*H* tetrazolium bromide (MTT, Acros Organics, Geel, Belgium) assay was used to detect the cytotoxicity of the compounds after 96-h incubation. For this purpose, cells were harvested from culture flasks by trypsinization, seeded in 100-μL aliquots into 96-well microculture plates (Starlab, UK) in densities of 1 × 10^3^ (CH1/PA-1), 2 × 10^3^ (SW480), and 3 × 10^3^ (A549) cells per well and incubated for 24 h prior to exposure to the test compounds. Stock solutions of test compounds were prepared in DMF, which were then diluted in MEM (not to exceed a final content of 0.5% v/v of organic solvent in the test plates), and serial dilutions were added in aliquots of 100 μL per well. After continuous exposure for 96 h, drug solutions were replaced with 100 μL medium/MTT mixtures [6 parts of RPMI 1640 medium supplemented with 10% heat-inactivated fetal bovine serum and 2 mM l-glutamine; 1 part of MTT solution in PBS (5 mg/mL)]. After incubation for 4 h, the medium/MTT mixtures were removed, and the produced formazan crystals were dissolved in 150 μL DMSO per well. Optical densities at 550 nm were measured spectrophotometrically with a ELx808 Absorbance Microplate Reader (Bio-Tek, Winooski, VT, USA) by using a reference wavelength of 690 nm to correct for unspecific absorption. 50% inhibitory concentrations (IC_50_) were interpolated from concentration−effect curves of at least three independent experiments, each comprising triplicates per concentration level.

### 3.5. Resazurin Assay

The resazurin assay was assessed for comparing cytotoxicities between 2D and 3D cell culture approaches of chosen compounds after 96-h incubation. For 2D experiments, HCT116 cells were harvested from culture flasks by trypsinization, seeded in 100-μL aliquots into 96-well microculture plates (CytoOne, TC-treated, from Starlab) in densities of 2 × 10^3^ cells per well, and incubated for 24 h prior to exposure to the test compounds. For 3D-model formation, single HCT116 cells were seeded in densities of 0.5 × 10^3^ in 100-μL aliquots in 96-well round-bottomed ultra-low attachment plates (Corning) and preincubated for 96 h in order to form spheroids before treatment with the substances. Stock solutions of test compounds were prepared in DMF, which were then diluted in McCoy's 5A-supplemented medium (not to exceed a final content of 0.5% v/v of organic solvent in the test plates), and serial dilutions were added in aliquots of 100 μL per well. After continuous exposure for 96 h, 20 μL of a 440 μM resazurin sodium salt (Alfa Aesar) solution in PBS was added. After incubation for 4 h, fluorescence was measured with a microplate reader (Bio-Tek, SynergyHT) at a wavelength of 530 nm, additionally using a reference wavelength of 620 nm. For 3D experiments, 20 μL of a 440 μM resazurin sodium salt solution was added after 24 h before the end of the 96-h exposure period. Fluorescence was measured (as above), and all 50% inhibitory concentrations (IC_50_) were interpolated from concentration−effect curves of at least three independent experiments.

### 3.6. DCFH-DA Assay

SW480 colon adenocarcinoma cells were trypsinized for 5 min in a humidified incubator at 37°C and under a 5% CO_2_ atmosphere. Supplemented MEM was added to stop trypsinization, and cells were centrifuged for 3 min at 1200 rpm (Heraeus Megafuge 1.0R). After cell counting, SW480 cells were seeded in 100-μL aliquots in densities of 2.5 × 10^4^ cells/well into 96-well clear flat-bottom microplates (CytoOne, TC-treated, from Starlab). After incubation for 24 h, cells were washed with 200 μL of Hanks' balanced salt solution (HBSS; Sigma-Aldrich; supplemented with 1% heat-inactivated FCS). Then, cells were incubated with 100 μL/well of 25 μM 2′,7′-DCFH-DA (in supplemented HBSS) for 45 min at 37°C. After washing cells with 200 μL of supplemented HBSS, serially diluted test compounds in phenol-red-free Opti-MEM (Gibco), supplemented with 1% heat-inactivated FCS, were added in 200-μl triplicates and tert-butyl hydroperoxide (TBHP) was applied as positive control. Immediately after addition of the test compounds, fluorescence (ex/em = 485/516 nm) was measured every 10 min for a total period of 2 h with a microplate reader (Bio-Tek, Synergy HT). Blank-corrected values were calculated relative to negative controls (treated with drug-free supplemented Opti-MEM) from three independent experiments.

### 3.7. Cellular Accumulation

Cellular accumulation of the compounds was studied based on a method described previously [48] with modifications. 1.8 × 10^5^ SW480 cells per well were seeded in aliquots of 1 mL of complete MEM (see above) into 12-well plates (CytoOne, tissue culture treated, Starlab) and incubated at 37°C for 24 h. Then, cells were exposed for 2 h at 37°C to 50 μM solutions of the test compounds (containing 0.5% DMF) in fresh 0.5 mL of complete MEM upon exchange of the medium. Afterward, cells were washed three times with 1 mL PBS per well, lysed with 0.4 mL sub-boiled HNO_3_ per well for 1 h at room temperature, and 0.3 mL of each sample was diluted with 7.7 mL Milli-Q water. Adsorption/desorption controls were prepared in the same manner in cell-free wells. Ruthenium content was quantified by ICP-MS using an ICP-quadrupole MS Agilent 7800 instrument (Agilent Technologies, Waldbronn, Germany) as described previously [49].

### 3.8. Stability in Aqueous Solution

The exact concentration of the metal complex stock solutions was calculated on weight-in-volume basis. Complexes were dissolved in water in 1–5 mM concentration.

#### 3.8.1. UV–Vis Spectrophotometric Measurements

An Agilent Cary 8453 diode array spectrophotometer and an Agilent Cary 3500 8-channel scanning photometer were used to record the UV–vis spectra in the interval 200–800 nm. The path length was 1 cm. Time-dependent assays were done for **3a** and **3c** in pH 1.0 (HCl), pH 2.0 (HCl), pH 7.4 (PBS or phosphate), pH 10 NaOH, and 3 M KCl applying 90–160 μM complex concentrations.

An Orion 710A pH meter equipped with a Metrohm combined electrode (Type 6.0234.100) and a Metrohm 665 Dosimat burette were used for the pH-dependent measurements of **3a** and **3c**. The electrode system was calibrated according to the method suggested by Irving et al. [50] The average water ionization constant, p*K*_w_, was determined as 13.76 ± 0.01, which is in good agreement with literature data [51]. The initial volume of the samples was 15.0 mL. The complex concentration was 100 or 157 μM at an ionic strength of *I = *0.1 M (KCl). Samples were degassed by bubbling purified argon through them for about 10 min prior to the measurements, and the inert gas was also passed over the solutions during the titrations. 0.70-mL batch samples were collected between pH 1 and 11; these samples were kept in dark, and after 2 and 3 days, their pH was remeasured, and the UV–vis spectra were recorded as well. Only small differences were observed between the 2- and 3-day spectra, and always, the 3-day samples were used for further evaluation. The conditional stability constant for **3c** was calculated based on the following equilibrium: [RuCym(N,C)(*Z*)]^(1+*n*)^ + MeIm ⇌ [RuCym(N,C) (MeIm)]^+^ + *Z*^*n*^; (*Z* = Cl^–^ or H_2_O; *n* = −1 or 0). The calculation was done with the HypSpec software [40]. The deprotonation constant of HMeIm was known from the literature for the same ionic strength (p*K*_a_ = 7.14, *I* = 0.1 M KCl) [36]. The calculated stability constant is a conditional value and applies for *I* = 0.1 M KCl ionic strength.

#### 3.8.2. ^1^H-NMR Measurements

^1^H-NMR spectroscopic studies were carried out on a Bruker FT-NMR spectrometer AV NEO 500.10 MHz and a Bruker Avance III HD Ascend 500 Plus instrument. All ^1^H-NMR spectra were recorded using a standard Bruker noesygppr1d pulse sequence to suppress water resonance. Stock solutions of **3a** and **3c** were made in water. Samples contained 10% (v/v) D_2_O with a complex concentration of ca. 1 mM. Samples were prepared in pH 1.3 aqueous HCl solution or in pH 7.4 PBS.

### 3.9. FRET Melting Assay

FRET experiments were conducted using an Applied Biosystems QuantStudio 6 PCR cycler in a 96-well format equipped with a FAM (6-carboxyfluorescein) filter. Oligonucleotide stock solutions, labelled with FAM and TAMRA (6-carboxytetramethylrhodamine) probes, were diluted to the desired concentration in a 60-mM potassium cacodylate buffer (pH 7.4). To fold the oligonucleotides into their B-DNA or G4 topologies, the solutions were heated to 95°C for 5 min and then allowed to cool slowly to room temperature overnight. The final concentration of the oligonucleotides was 0.2 μM, with a total volume of 30 μL in each well. Metal complexes were dissolved in DMF to prepare 2 mM stock solutions and further diluted with the buffer, where DMF concentration never exceeded 0.05%. Mixtures of oligonucleotides and metal compounds were left at room temperature for 2 h. Data were collected twice, each time in duplicate, over a temperature range of 25°C–95°C, with a ramp of 1°C every 30 s. FAM emission data were normalized from 0 to 1 to compare different datasets. After data normalization, we extrapolated the T_1/2_ values, i.e., the temperature at which normalized FAM emission is 0.5. The DNA concentration is expressed in strands.

### 3.10. Animal Studies

All experiments were approved by the Ethics Committee for the Care and Use of Laboratory Animals at the Medical University Vienna (proposal number 2022-0.770.291) and performed according to the guidelines from the Austrian Animal Science Association and from the Federation of European Laboratory Animal Science Associations (FELASA). All animals were kept in a pathogen-free environment with a 12 h light dark cycle with *ad libitum* access to food and water. Every procedure was performed in a laminar airflow under sterile conditions.

### 3.11. Allograft In Vivo Experiments in CT-26-Bearing BALB/c Mice

CT26 cells (5 × 10^5^ cells in 50-μL serum-free medium) were injected subcutaneously (s.c.) into the right flank of 8–16-week-old BALB/c mice (Envigo Laboratories, San Pietro Al Natisone, Italy). After 3 days, therapy treatment started on five consecutive days for 2 weeks. Animals were treated i.p. with 50 mg/kg **1c** and **3c** (all dissolved in 10% DMSO in 0.9% NaCl, 12 mice per group). The solvent control animals received 10% DMSO in 0.9% NaCl. Every day, the animals were monitored for the overall health conditions and tumor size was measured regularly by caliper measurement. Tumor volumes (mm^3^) were calculated using following formula: length × width^2^/2. In the overall survival arm of the experiment, mice were sacrificed by cervical dislocation in the case of a tumor length > 20 mm or tumor ulceration or a decreased body weight of ∼20%. Tumor growth and possible side effects of the treatment were evaluated by daily recording the tumor size by caliper measurement and parameters of the animal's overall health conditions. In the tissue sampling arm at the experiment, the mice were sacrificed by cervical dislocation 2 h after the last treatment. Tumors as well as organs were collected. Additionally, blood was drawn and incubated for 25 min to allow blood clotting. To isolate serum and blood pellets, the blood was centrifuged for 10 min at 17,900 g at room temperature. The supernatant characterized as serum was transferred to a new tube and centrifuged again to remove residual red blood cells. All collected samples were stored at −20°C and further processed for platinum measurements via ICP-MS.

### 3.12. Measurement of Ruthenium Level in Organs via ICP-MS

All tissue samples were digested (approx. 15–30 mg gravimetrically weighted) with 2 mL of approx. 20% nitric acid (69%, Rotipuran, supra-pure for trace metal analysis, NORMATOM; Distributor: VWR international, Austria) and 100 μL H_2_O_2_ (conc. H_2_O_2_ supra-pure (30%)) using an open vessel graphite digestion system (coated graphite heating plate, coated sample holder-top for 25 mL vials, PFA vials, and PFA lids; Labter, ODLAB; Distributor: AHF Analysentechnik AG; Germany). Samples were diluted in Milli-Q water (18.2 MΩ cm, Milli-Q Advantage, Darmstadt, Germany). The ruthenium concentration was determined by ICP-MS analysis. Ruthenium and rhenium standards were derived from LabKings (LabKings B.V., The Netherlands). The total ruthenium content was determined with a quadrupole-based ICP-MS instrument Agilent 7800 (Agilent Technologies, Tokyo, Japan) equipped with the Agilent SPS 4 autosampler (Agilent Technologies, Tokyo, Japan) and a MicroMist nebulizer at a sample uptake rate of approximately 0.2 mL·min^−1^. A radio frequency power of 1550 W was used as well as nickel cones. Argon was used as plasma gas (15 L·min^−1^) and as carrier gas (∼1.08 L·min^−1^). The integration time was set to 0.1 s, and measurements were performed in 6 replicates with 100 sweeps. Rhenium served as internal standard for ruthenium. The Agilent MassHunter software package (Workstation Software, Version C.01.06, 2019) was used for data processing.

## Figures and Tables

**Table 1:** Inhibition of cancer cell growth in three human cancer cell lines, determined by the MTT assay (exposure time: 96 h).

	Compound	A549 (μM)	SW480 (μM)	CH1 (μM)
Ru	**3a** **1a** [5]	11 ± 1 24 ± 4^∗^	5.4 ± 1.1 9.5 ± 0.7^∗^	1.9 ± 0.1 3.0 ± 0.9^∗^
	**3b** **1b** [5]	20 ± 3 16 ± 1^∗^	7.9 ± 1.5 8.6 ± 0.9^∗^	2.8 ± 0.2 3.4 ± 0.2^∗^
	**3c** **1c** [5]	> 200 12 ± 1^∗^	> 200 5.1 ± 0.4^∗^	102 ± 11 1.6 ± 0.4^∗^
	**3d** **1d** [5]	7.2 ± 1.0 34 ± 3^∗^	3.8 ± 0.3 19 ± 2^∗^	1.61 ± 0.04 14 ± 2^∗^
	**3e** **1e** [5]	14 ± 1 27 ± 1^∗^	5.5 ± 2.2 17 ± 1^∗^	1.8 ± 0.3 7.3 ± 0.4^∗^

Os	**4a** **2a** [5]	12 ± 1 17 ± 1^∗^	5.9 ± 1.9 9.3 ± 1.1^∗^	2.3 ± 0.2 3.0 ± 0.2^∗^
	**4b** **2b** [5]	20 ± 2 10 ± 1^∗^	7.4 ± 2.3 7.1 ± 0.3^∗^	2.7 ± 0.2 2.0 ± 0.4^∗^
	**4c** **2c** [5]	> 200 10 ± 1^∗^	> 200 4.4 ± 0.5^∗^	> 200 0.83 ± 0.14^∗^
	**4d** **2d** [5]	7.1 ± 0.6 17 ± 1^∗^	3.4 ± 0.9 9.3 ± 1.6^∗^	1.2 ± 0.2 3.7 ± 0.6^∗^
	**4e** **2e** [5]	24 ± 1 14 ± 1^∗^	8.1 ± 2.6 7.1 ± 1.2^∗^	2.1 ± 0.5 2.2 ± 0.3^∗^

*Note:* 50% inhibitory concentrations (means ± standard deviations) from at least three independent experiments.

^∗^IC_50_ values of the chlorido complexes were added as reference [5].

**Table 2:** Cellular accumulation of compounds **3a–e** (50 μM, 0.5% DMF in MEM) in SW480 cancer cells (exposure time: 2 h) and calculated logP (clogP) values of the corresponding free ligands.

Compound	fg Ru/cell	clogP (free ligand)	Solubility (mM)
**3a**	387 ± 16	2.54	9.08
**3b**	470 ± 71	2.71	8.82
**3c**	4.6 ± 0.6	1.41	24.6
**3d**	477 ± 47	2.99	8.92
**3e**	246 ± 47	2.60	15.6

**Table 3:** Cytotoxic activity of selected compounds in 2D and 3D cultures of the HCT116 colorectal cancer cell line, determined by the fluorimetric resazurin assay after 96 h of exposure.

Compound	IC_50_ 2D (μM)	IC_50_ 3D (μM)	3D/2D
**1a**	18 ± 5	31 ± 5	1.7
**1c**	6.5 ± 1.3	8.9 ± 2.1	1.4
**3a**	8.4 ± 0.7	10.6 ± 0.9	1.3
**3c**	> 200	226 ± 36	< 1.1
**4a**	9.6 ± 1.2	9.4 ± 4.6	0.98
**4c**	> 200	344 ± 19	< 1.7

*Note:* IC_50_ values (means ± standard deviations) were determined from interpolated concentration–effect curves of at least three independent experiments.

## Data Availability

The data that support the findings of this study are available in the supporting information of this article.

## Supporting Information

Electronic supporting information (ESI) available: ^1^H and ^13^C NMR spectra, mass spectra, X-ray diffraction analysis of CCDC 2384985 and 2384986, stability in aqueous solution, correlation IC_50_/cellular accumulation/clog*P*, ROS data, and G-quadruplex interaction studies.
