# Supplementary material for: Exploring the Anticancer Properties of 4-Phenylthiazole-Based Ru(II) and Os(II) Metallacycles Featuring 1-Methylimidazole as N-Donor Functionality
Source: Bioinorg Chem Appl. 2025 Sep 18;2025:6352081. doi: 10.1155/bca/6352081 (PMC12463534; doi:10.1155/bca/6352081)
Supplement: Supporting Information — Additional supporting information can be found online in the Supporting Information section. [file 6352081.f1.docx]

Supporting Information

Exploring the anticancer properties of 4-phenylthiazole-based Ru(II) and Os(II) metalacycles featuring 1-methylimidazole as *N*-donor functionality

Paul Getreuer^a,b^, Theresa Mendrina^a,c,d^, Steven van Terwingen^a^, Laura Marretta^e^, Orsolya Dömötör^f^, Dominik Wenisch^a^, Michaela Hejl^a^, Petra Heffeter^c,d^, Walter Berger^c,d^, Michael A. Jakupec^a,d^, Alessio Terenzi^e^, Bernhard K. Keppler^a,d^, and Wolfgang Kandioller^a,d,*^

^a^University of Vienna, Faculty of Chemistry, Institute of Inorganic Chemistry, Währinger Str. 42, 1090 Vienna, Austria,

^b^University of Vienna, Faculty of Chemistry, Vienna Doctoral School in Chemistry (DoSChem), Währinger Str. 42, 1090 Vienna, Austria,

^c^Medical University of Vienna, Center of Cancer Research and Comprehensive Cancer Center, Borschkegasse 8a, 1090 Vienna, Austria,

^d^Research Cluster “Translational Cancer Therapy Research”, Währinger Str. 42, 1090 Vienna, Austria,

^e^University of Palermo, STEBICEF-Department, Viale delle Scienze, Ed. 17, 90128 Palermo, Italy,

^f^University of Szeged, Department of Molecular and Analytical Chemistry, Interdisciplinary Excellence Centre, Dóm tér 7-8, 6720 Szeged, Hungary.

^*^wolfgang.kandioller@univie.ac.at, +43-1-4277-52609

**Table of Contents**

[1. ^1^H and ^13^C NMR spectra 2](#_Toc207892362)

[2. Mass spectra 12](#_Toc207892363)

[3. X-ray diffraction data 22](#_Toc207892364)

[4. Antiproliferative activity 25](#_Toc207892365)

[5. Stability in aqueous solution 27](#_Toc207892366)

[6. Cellular Accumulation 29](#_Toc207892367)

[7. ROS investigation 30](#_Toc207892368)

[8. G-Quadruplex interaction 31](#_Toc207892369)

[9. References 32](#_Toc207892370)

# ^1^H and ^13^C NMR spectra

## [((3-κN)-1-Methylimidazol)(4-phenylthiazolato-κN,κC2´)(η^6^-*p*-cymene)ruthenium(II)] nitrate (2a)


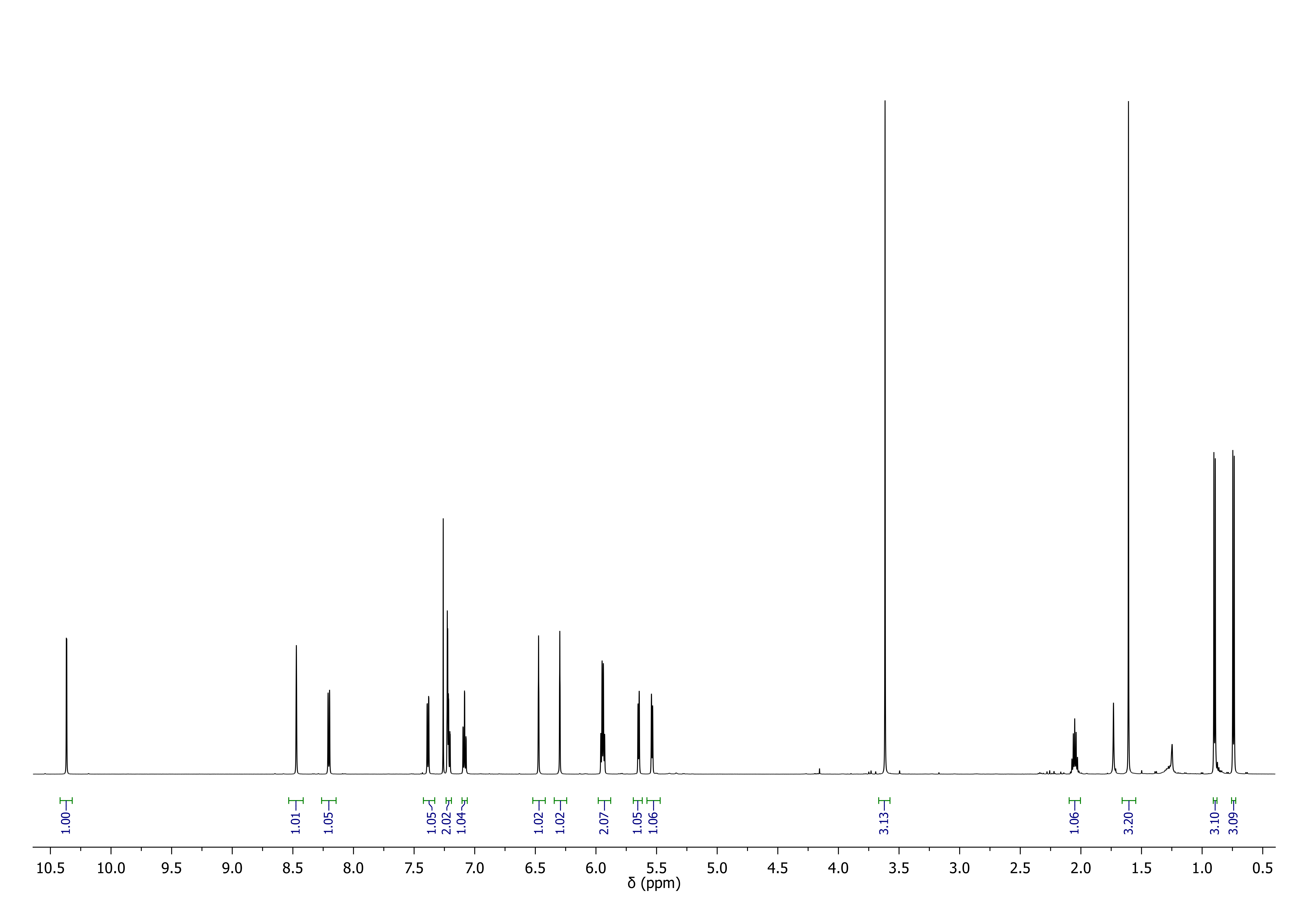


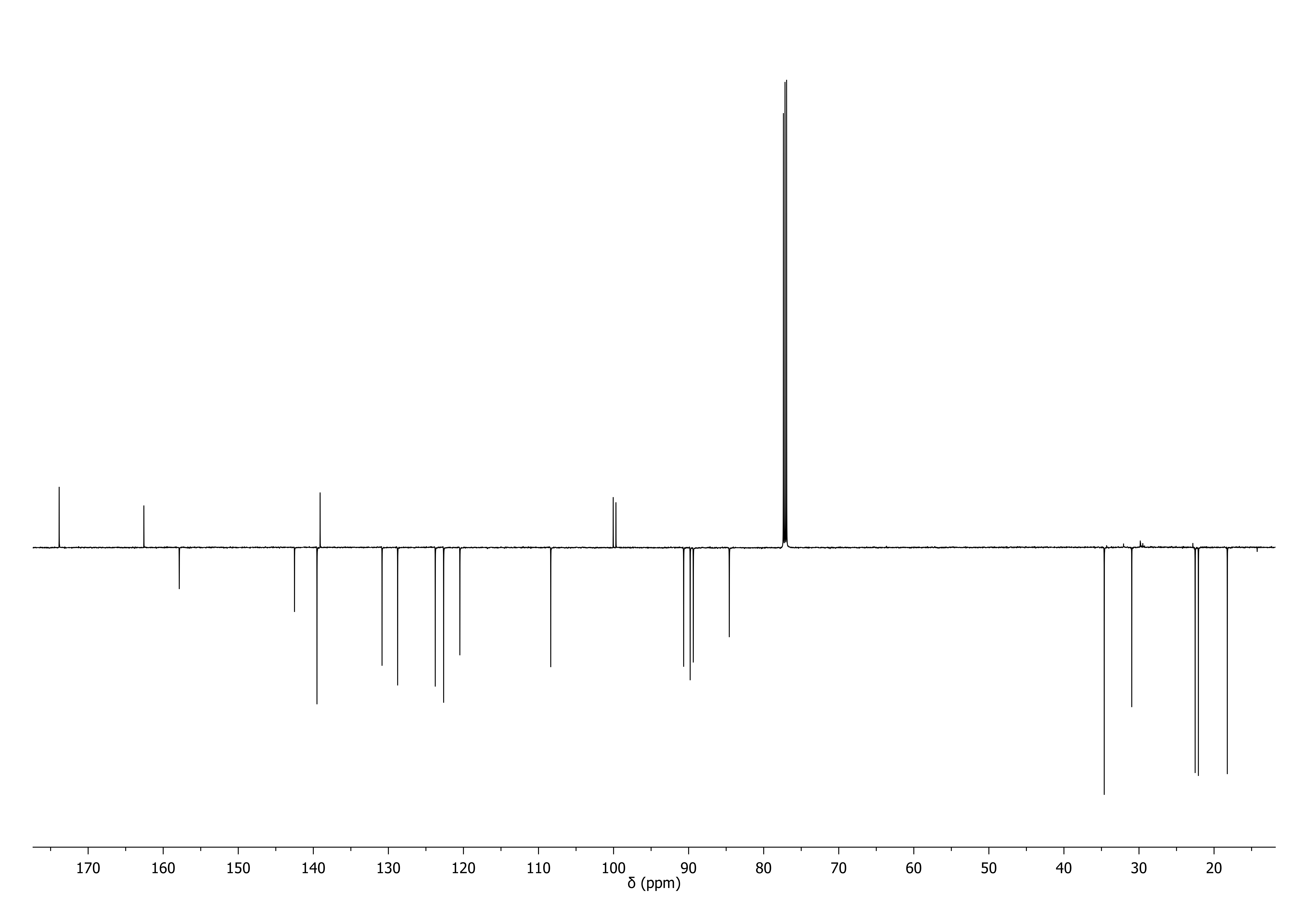


**Figure S1:** Top: Atom labelling and ^1^H-NMR spectrum of **2a**; Bottom: ^13^C-NMR spectrum of **2a**.

## [((3-κN)-1-Methylimidazol)(4-(4-fluorophenyl)thiazolato-κN,κC2´)(η^6^-*p*-cymene)ruthenium(II)] nitrate (2b)


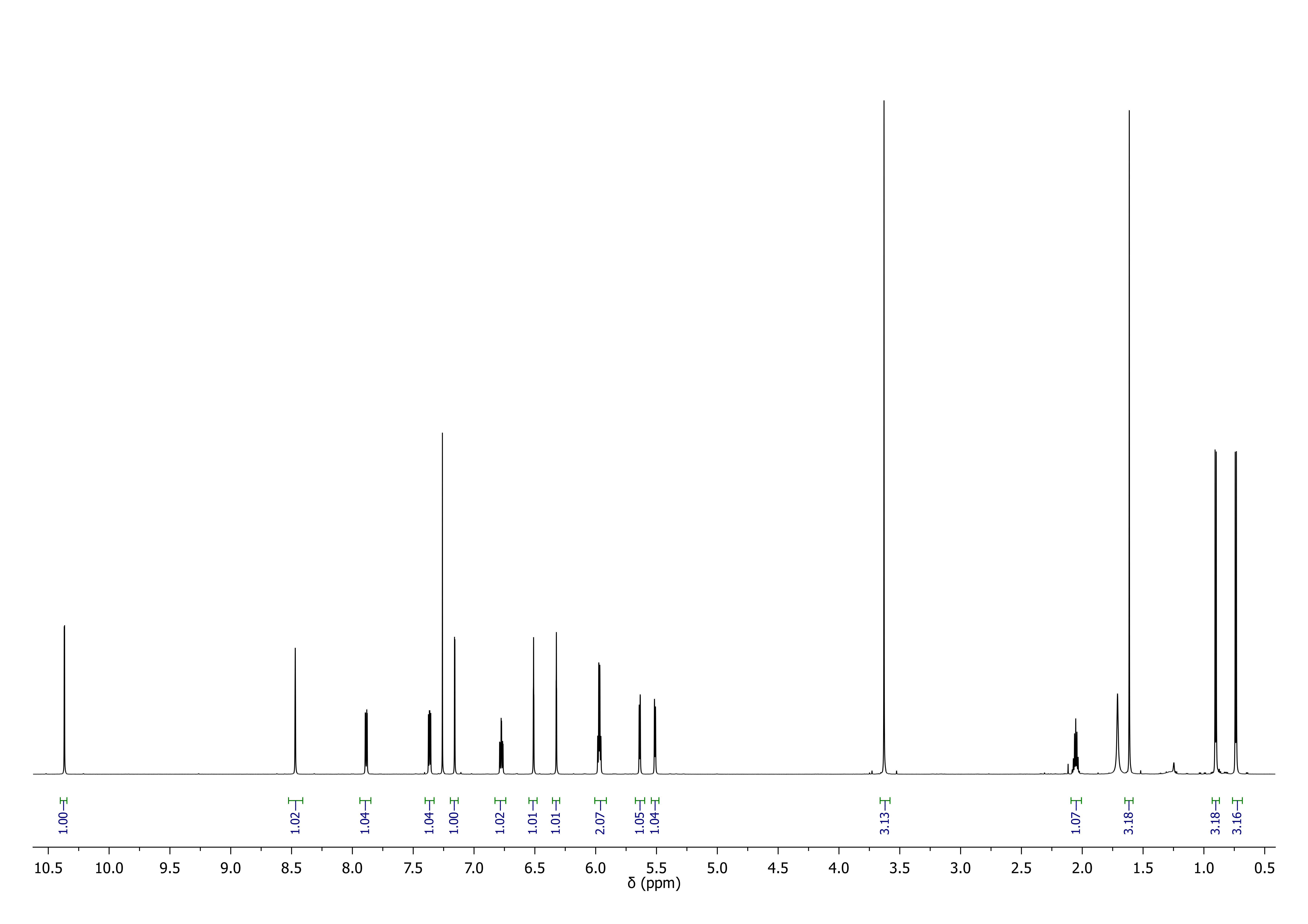

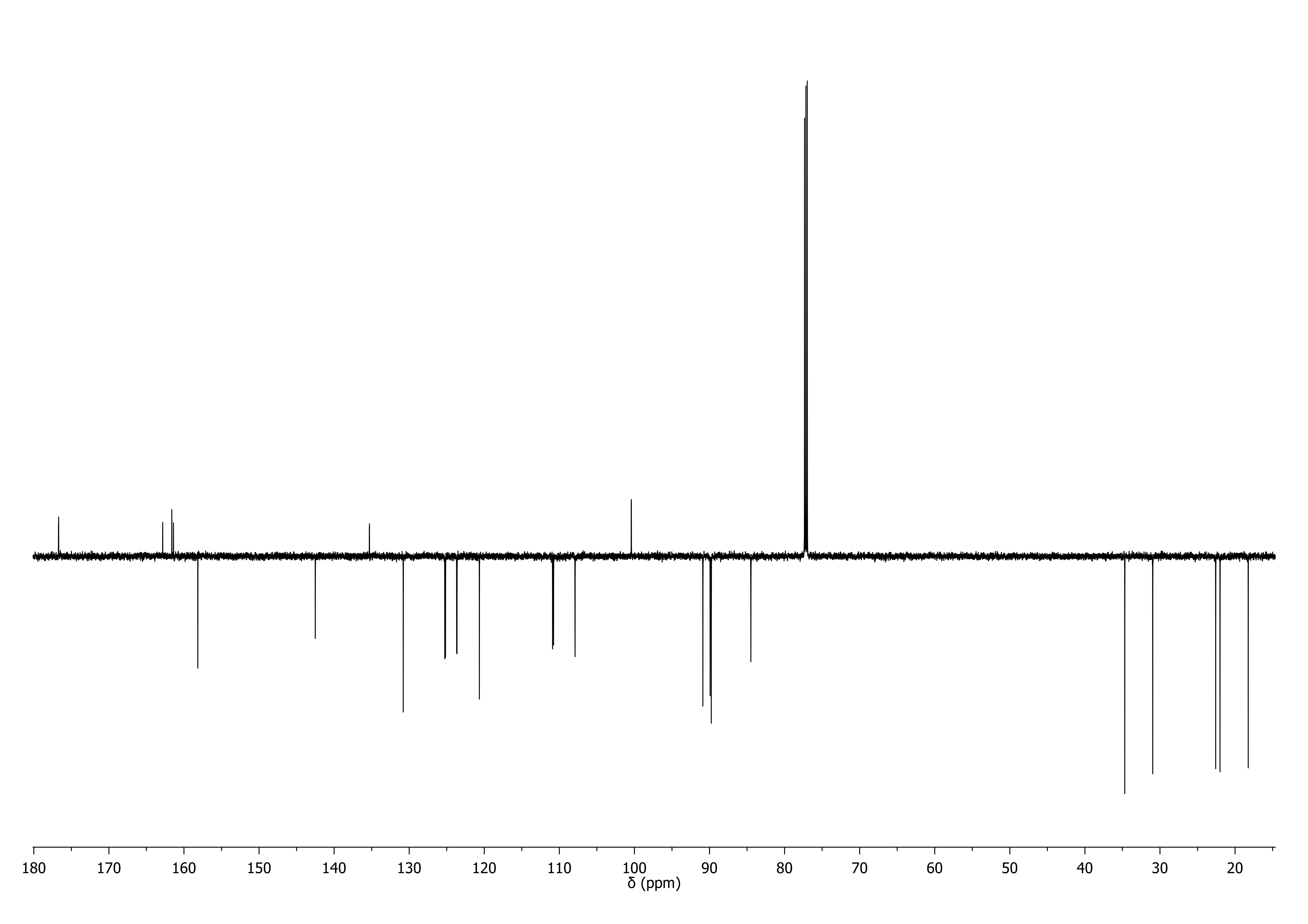


**Figure S2:** Top: Atom labelling and ^1^H-NMR spectrum of **2b**; Bottom: ^13^C-NMR spectrum of **2b**.

## [((3-κN)-1-Methylimidazol)(4-(4-(methylsulfonyl)phenyl)thiazolato-κN,κC2´)(η^6^-*p*-cymene)ruthenium(II)] nitrate (2c)


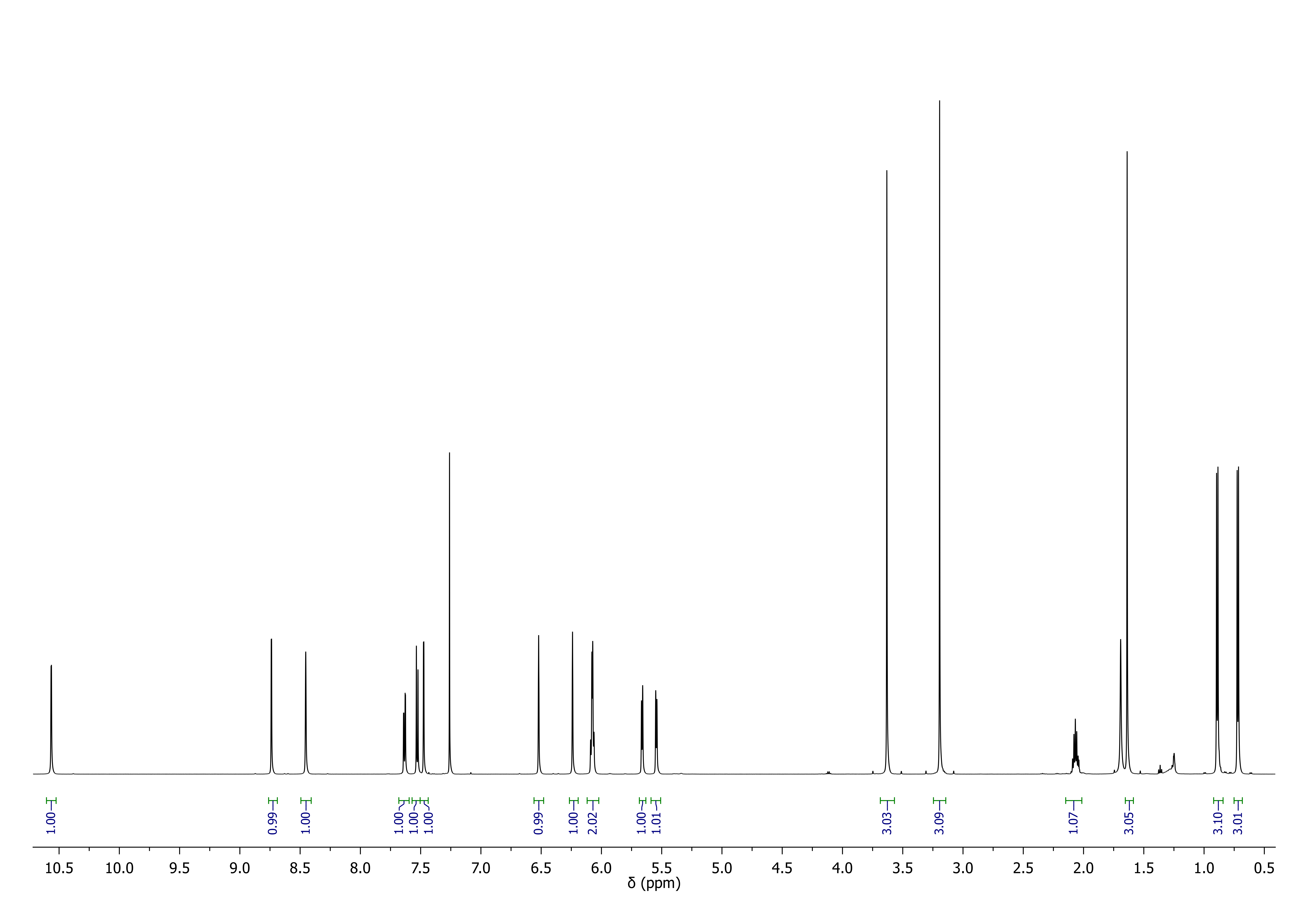

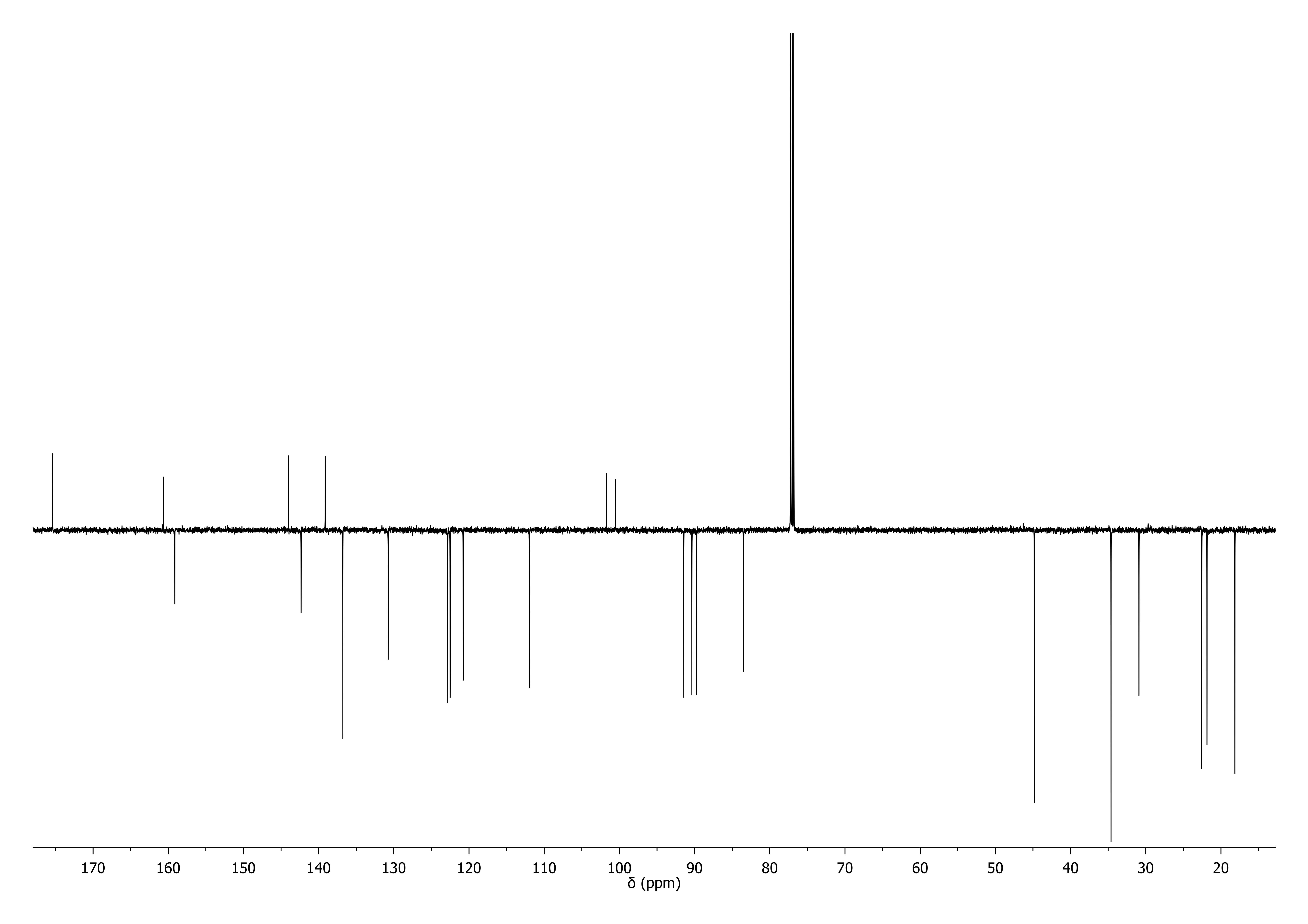


**Figure S3**: Top: Atom labelling and ^1^H-NMR spectrum of **2c**; Bottom: ^13^C-NMR spectrum of **2c**.

## [((3-κN)-1-Methylimidazol)(4-(4-methylphenyl)thiazolato-κN,κC2´)(η^6^-*p*-cymene)ruthenium(II)] nitrate (2d)


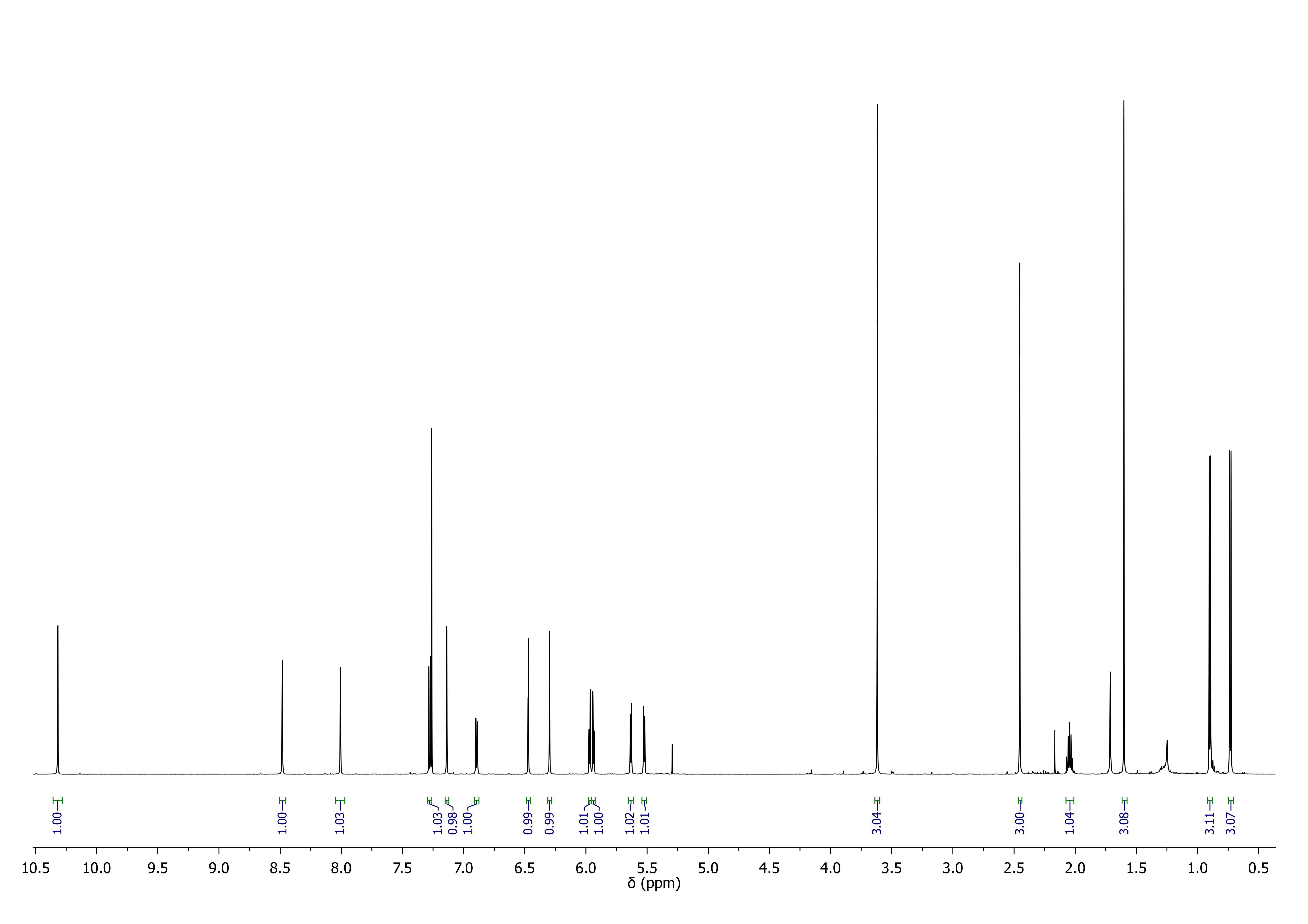

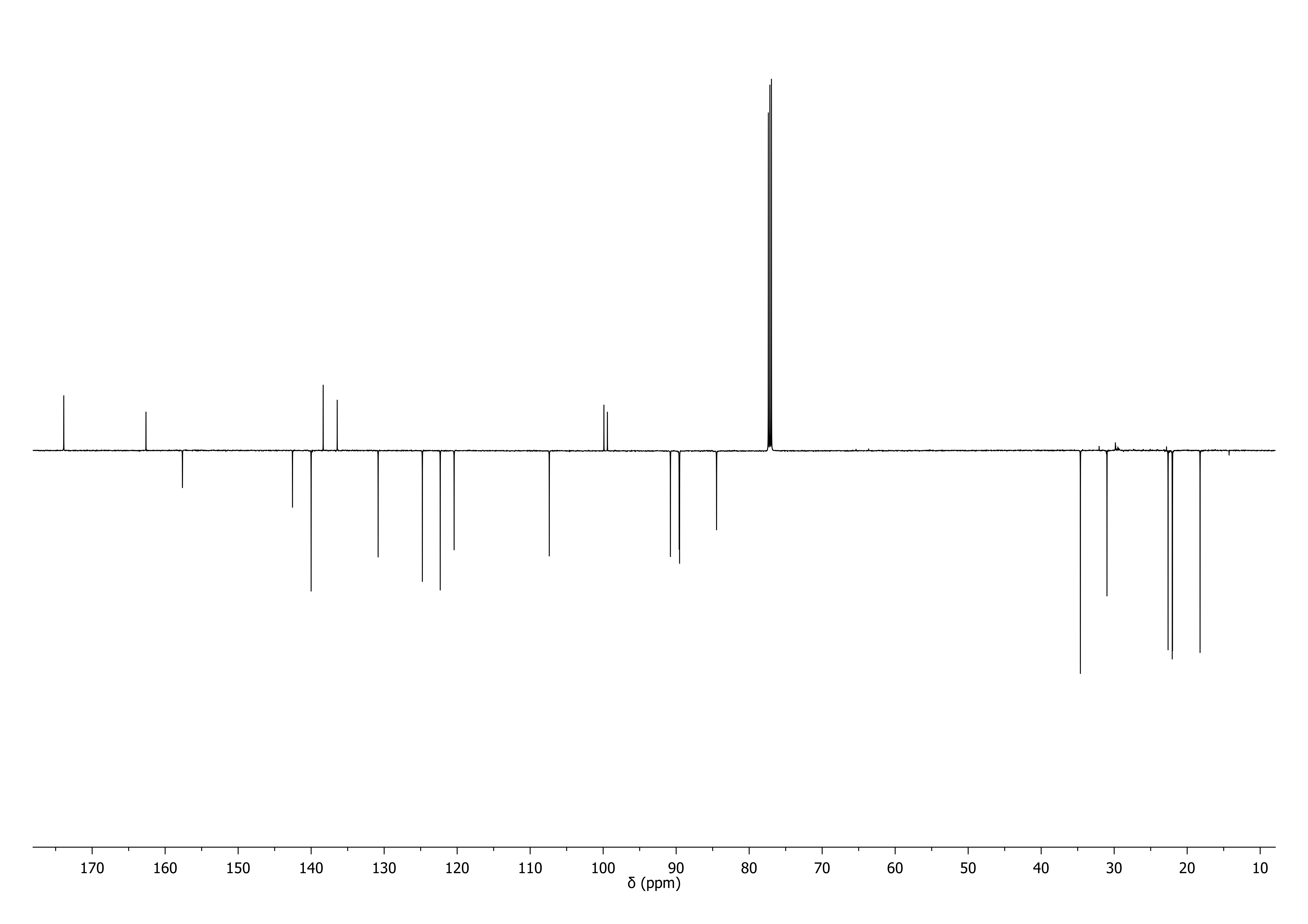


**Figure S4:** Top: Atom labelling and ^1^H-NMR spectrum of **2d**; Bottom: ^13^C-NMR spectrum of **2d**.

## [((3-κN)-1-Methylimidazol)(4-(4-methoxyphenyl)thiazolato-κN,κC2´)(η^6^-*p*-cymene)ruthenium(II)] nitrate (2e)


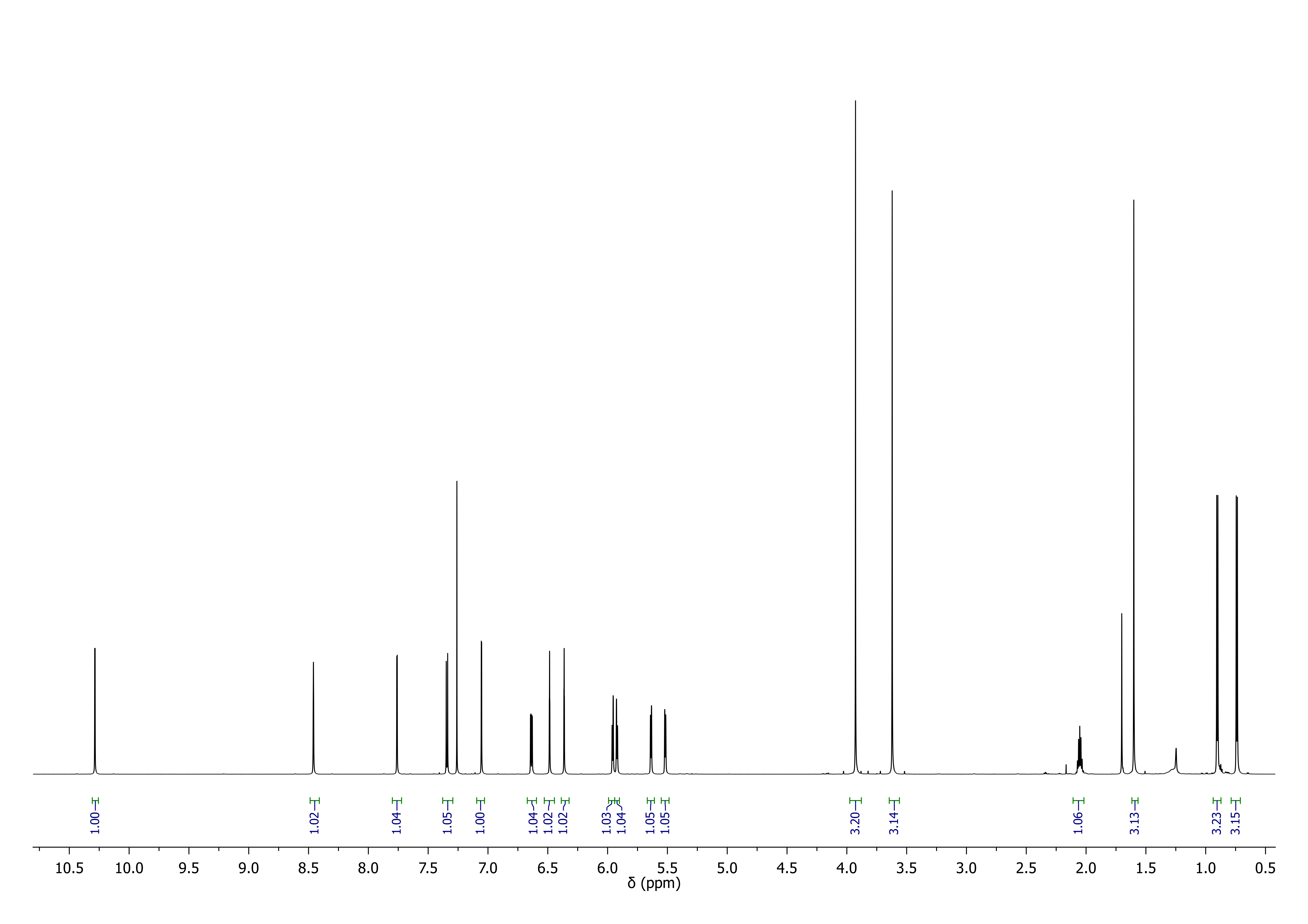

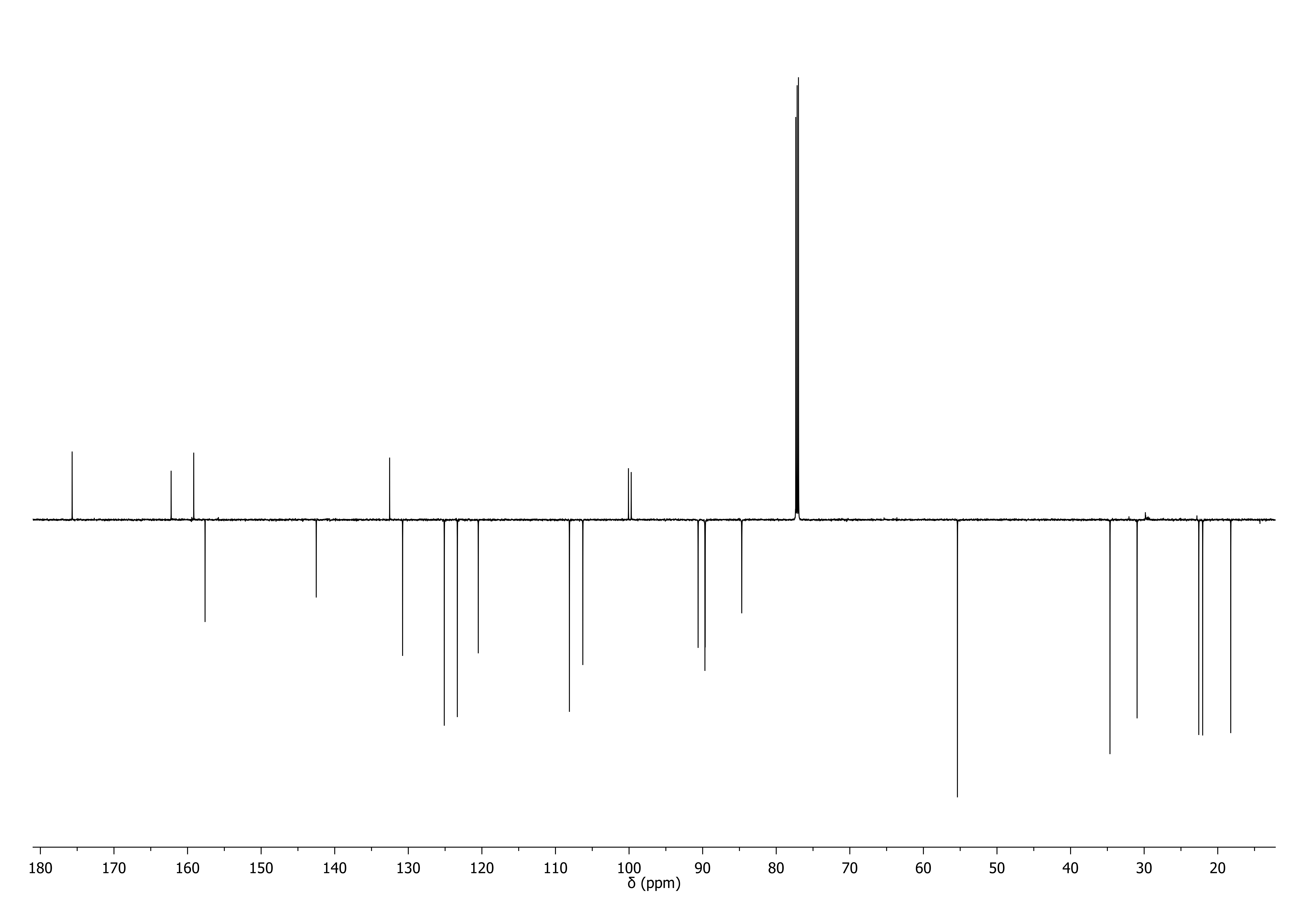


**Figure S5:** Top: Atom labelling and ^1^H-NMR spectrum of **2e**; Bottom: ^13^C-NMR spectrum of **2e**.

## [((3-κN)-1-Methylimidazol)(4-phenylthiazolato-κN,κC2´)(η^6^-*p*-cymene)osmium(II)] nitrate (4a)


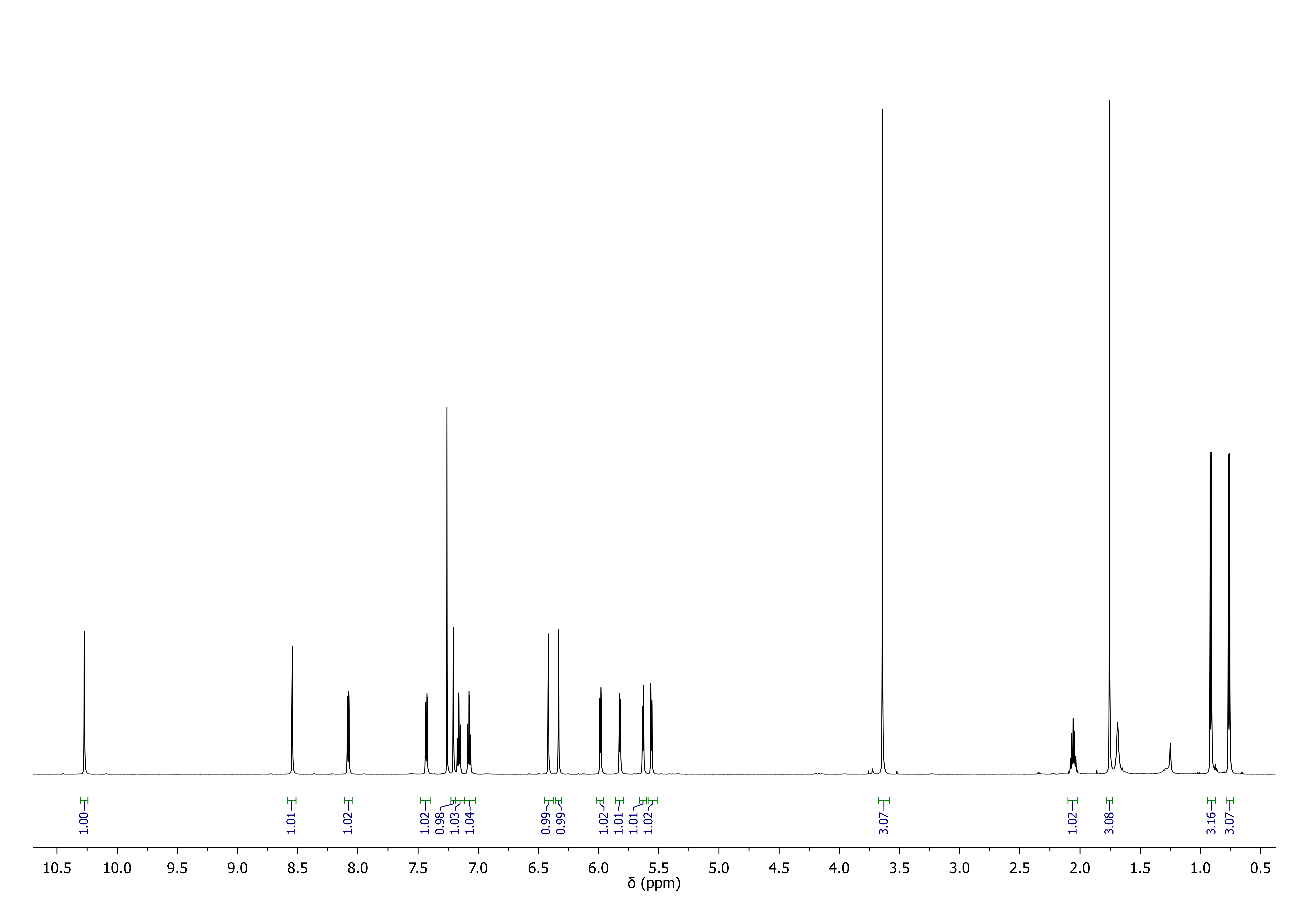

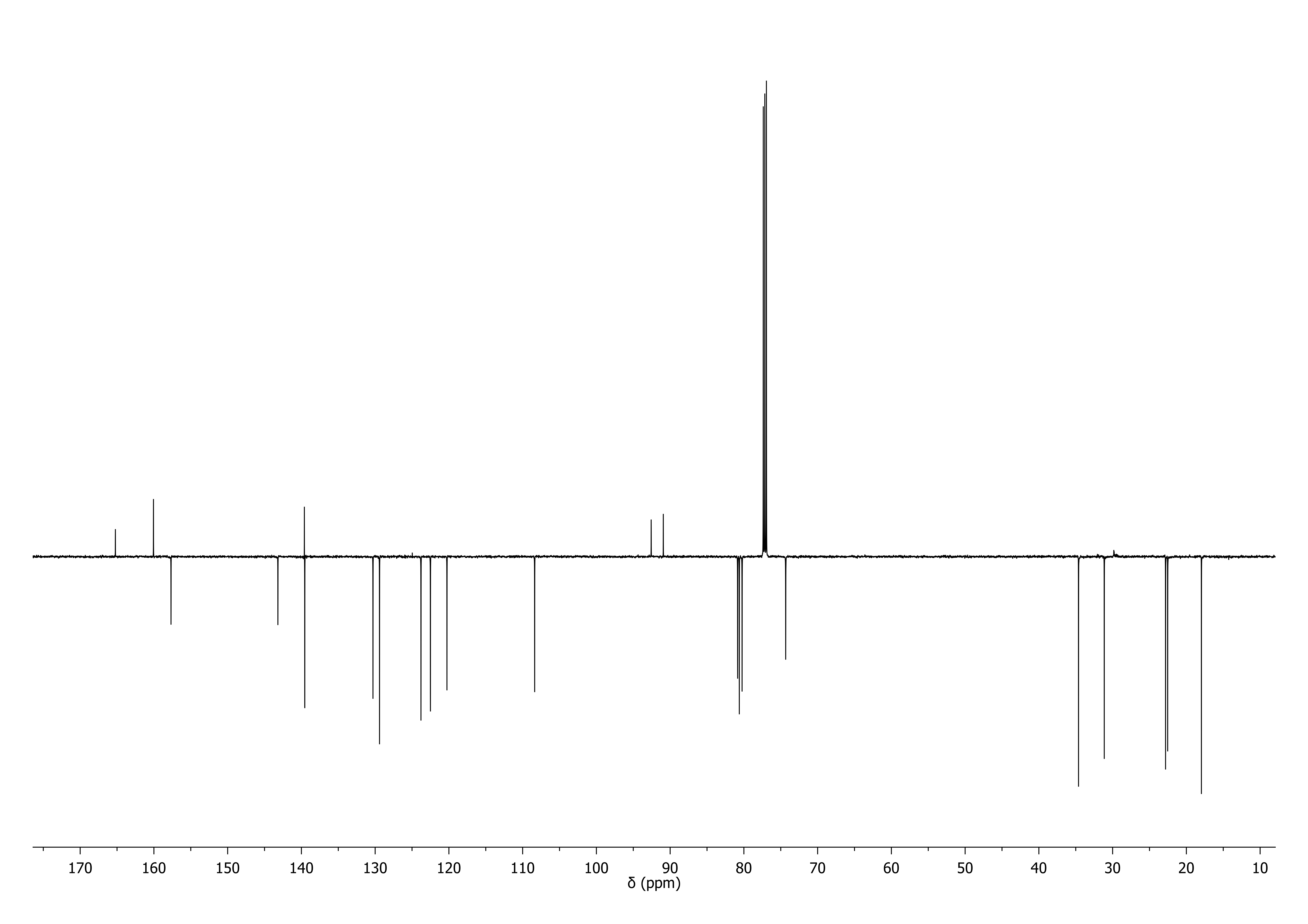


**Figure S6**: Top: Atom labelling and ^1^H-NMR spectrum of **4a**; Bottom: ^13^C-NMR spectrum of **4a**.

## [((3-κN)-1-Methylimidazol)(4-(4-fluorophenyl)thiazolato-κN,κC2´)(η^6^-*p*-cymene)osmium(II)] nitrate (4b)


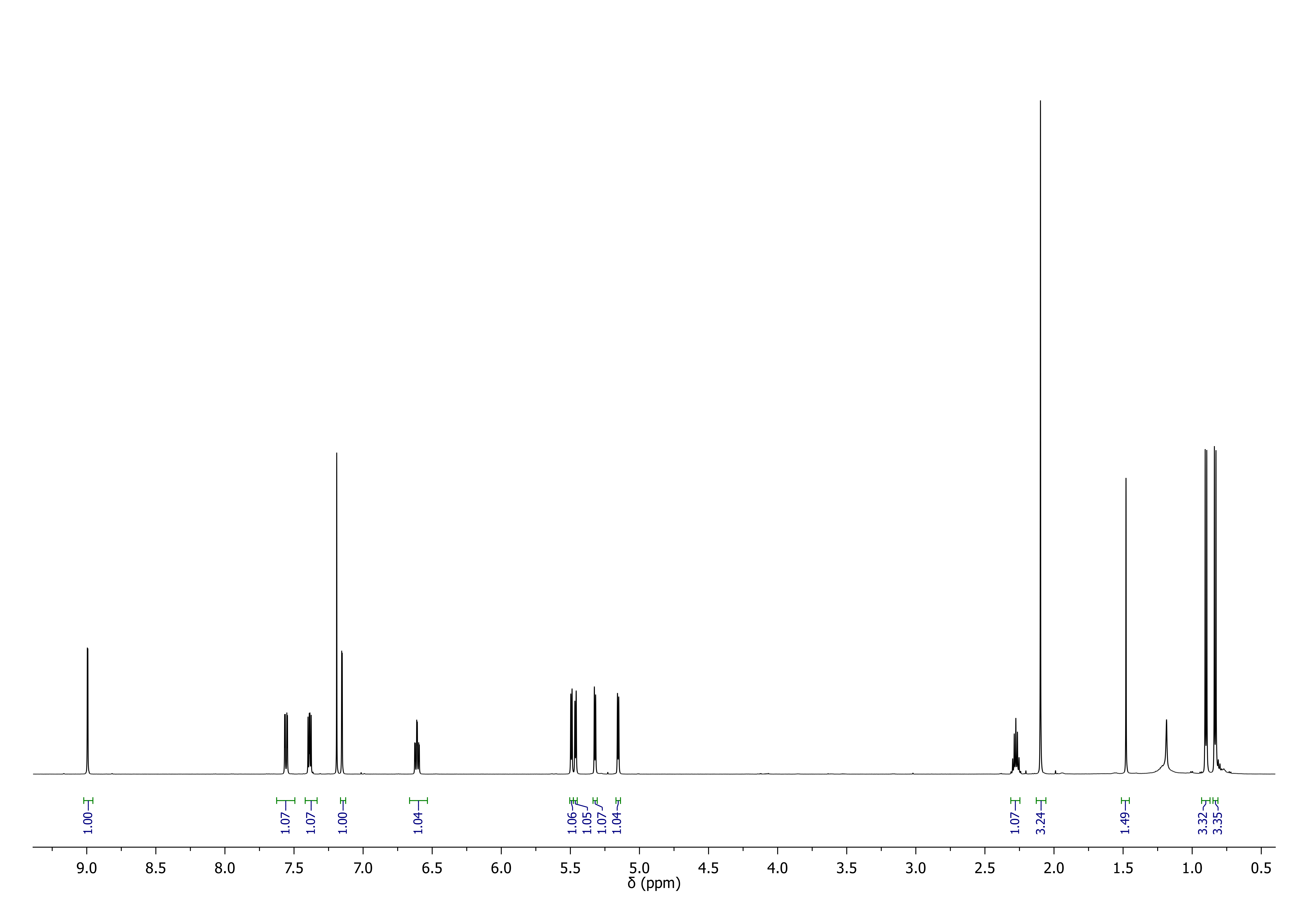

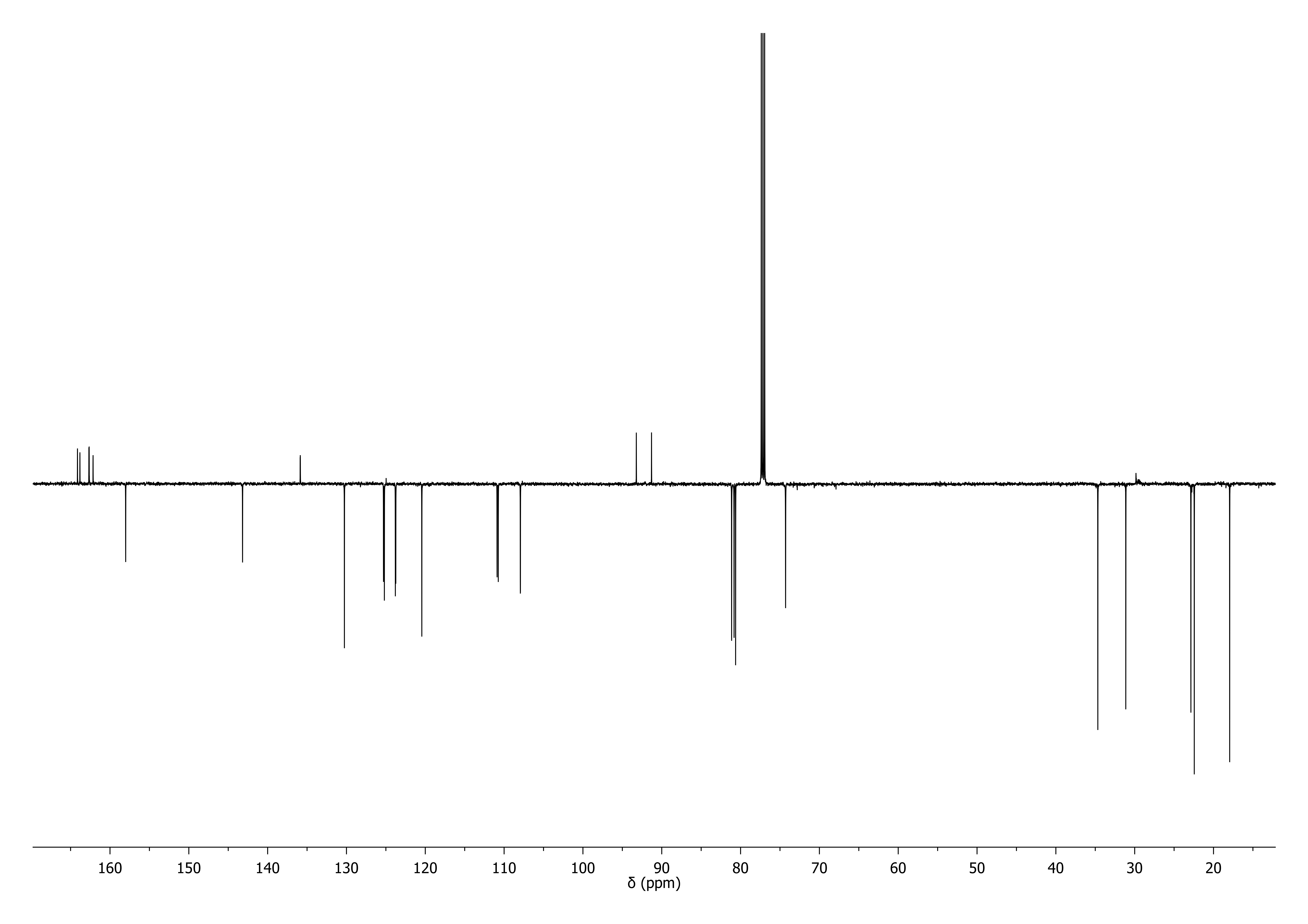


**Figure S7:** Top: Atom labelling and ^1^H-NMR spectrum of **4b**; Bottom: ^13^C-NMR spectrum of **4b**.

## [((3-κN)-1-Methylimidazol)(4-(4-(methylsulfonyl)phenyl)thiazolato-κN,κC2´)(η^6^-*p*-cymene)osmium(II)] nitrate (4c)


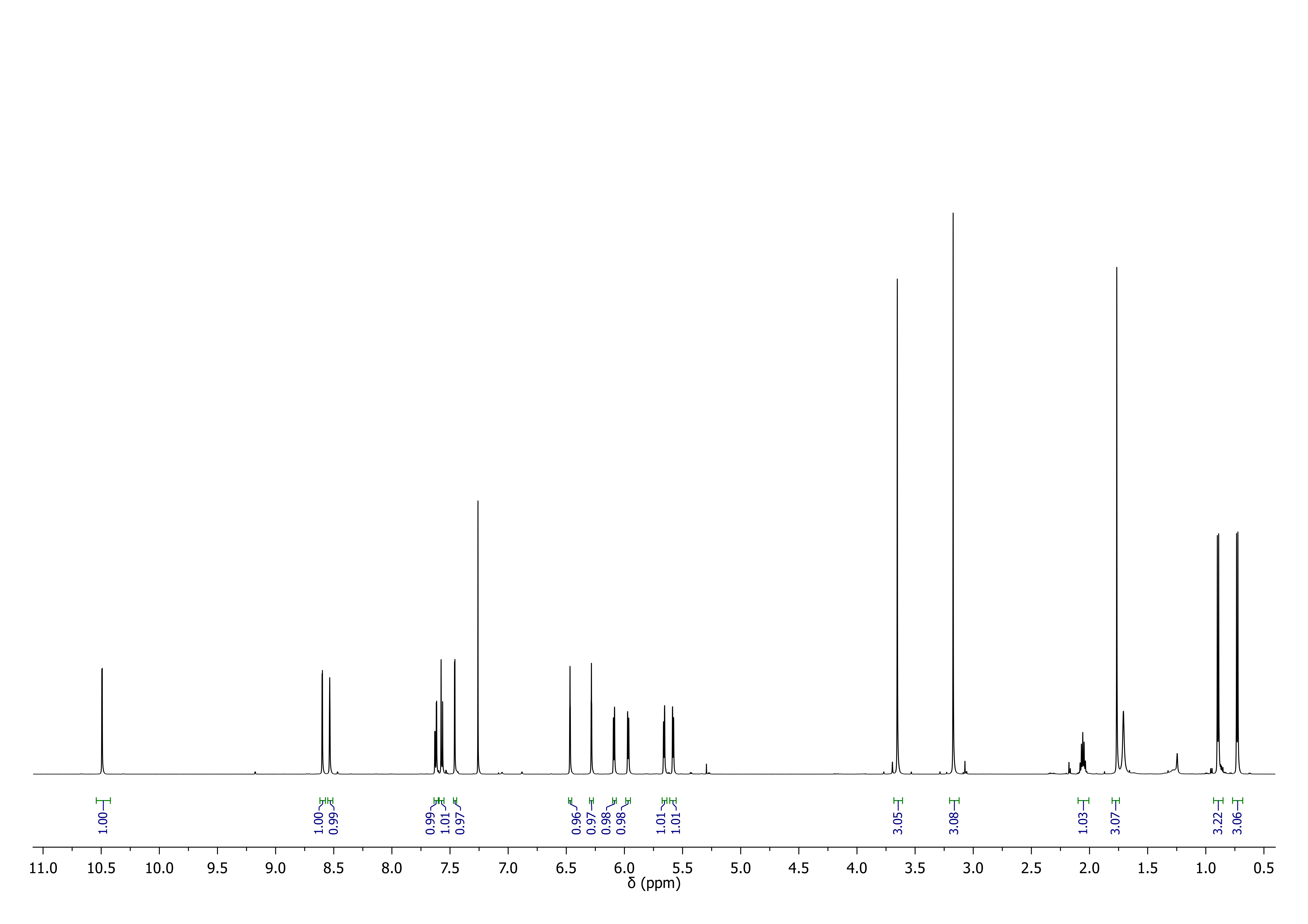

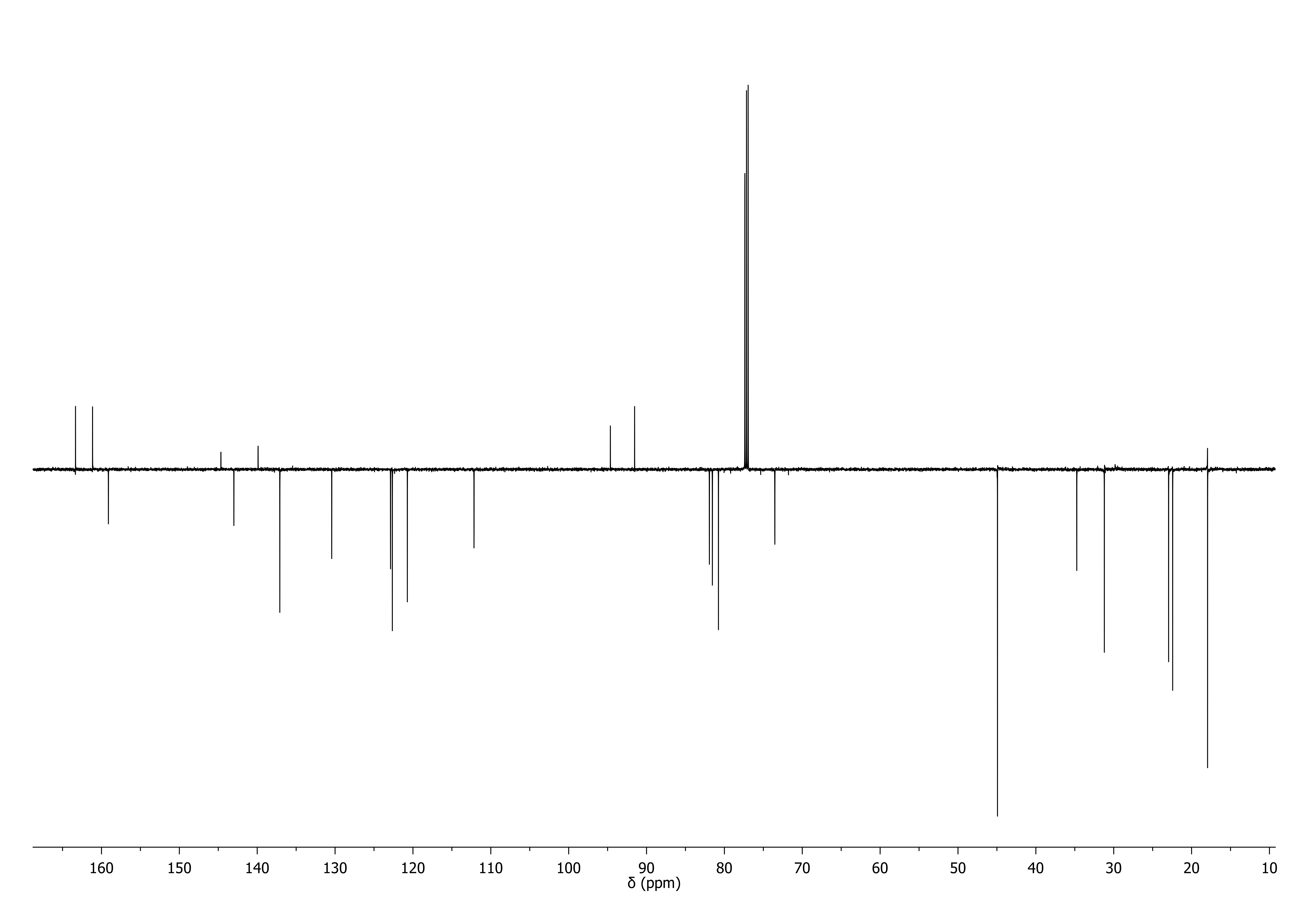


**Figure S8:** Top: Atom labelling and ^1^H-NMR spectrum of **4c**; Bottom: ^13^C-NMR spectrum of **4c**.

## [((3-κN)-1-Methylimidazol)(4-(4-methylphenyl)thiazolato-κN,κC2´)(η^6^-*p*-cymene)osmium(II)] nitrate (4d)


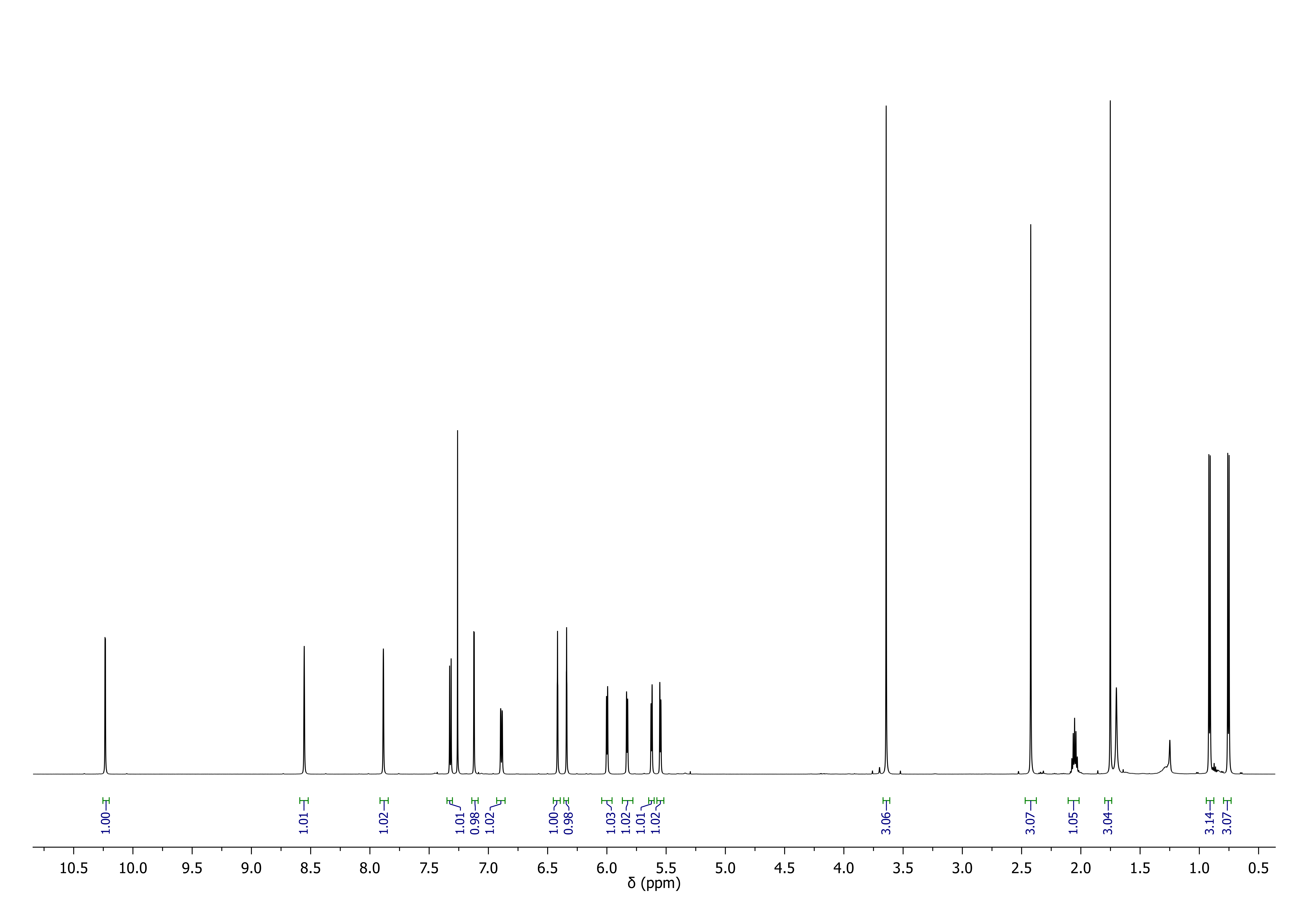

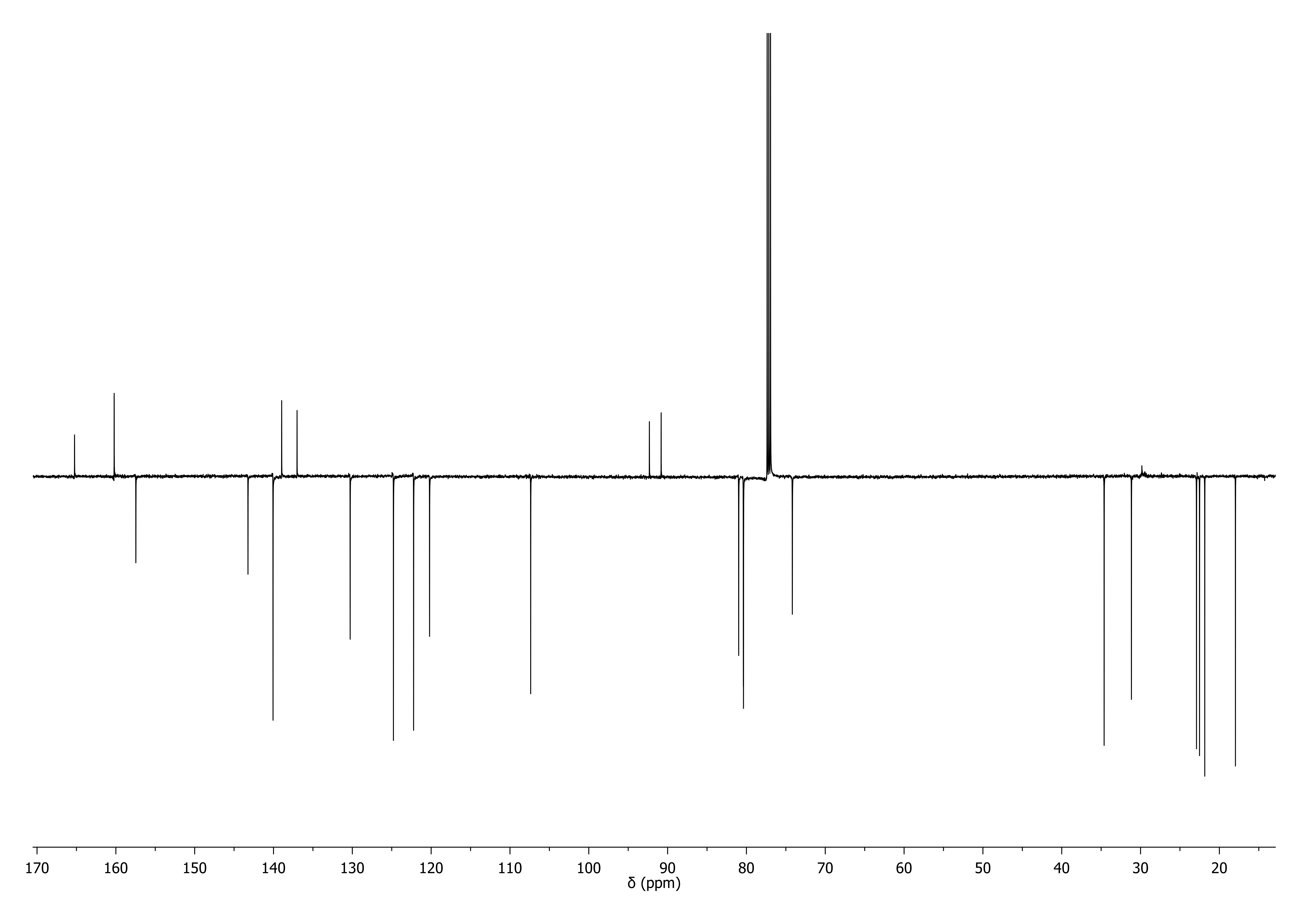


**Figure S9:** Top: Atom labelling and ^1^H-NMR spectrum of **4d**; Bottom: ^13^C-NMR spectrum of **4d**.

## [((3-κN)-1-Methylimidazol)(4-(4-methoxyphenyl)thiazolato-κN,κC2´)(η^6^-*p*-cymene)osmium(II)] nitrate (4e)


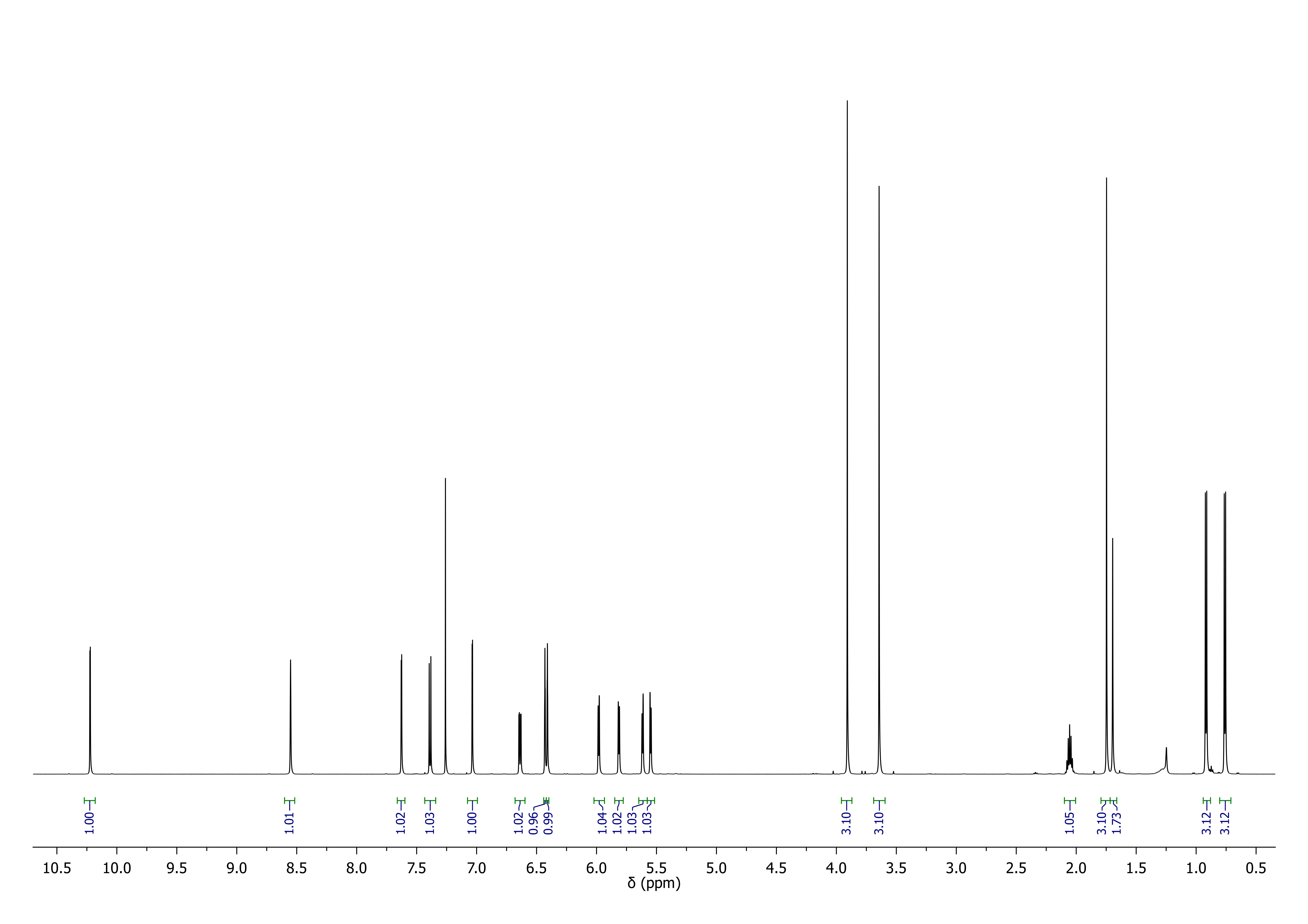

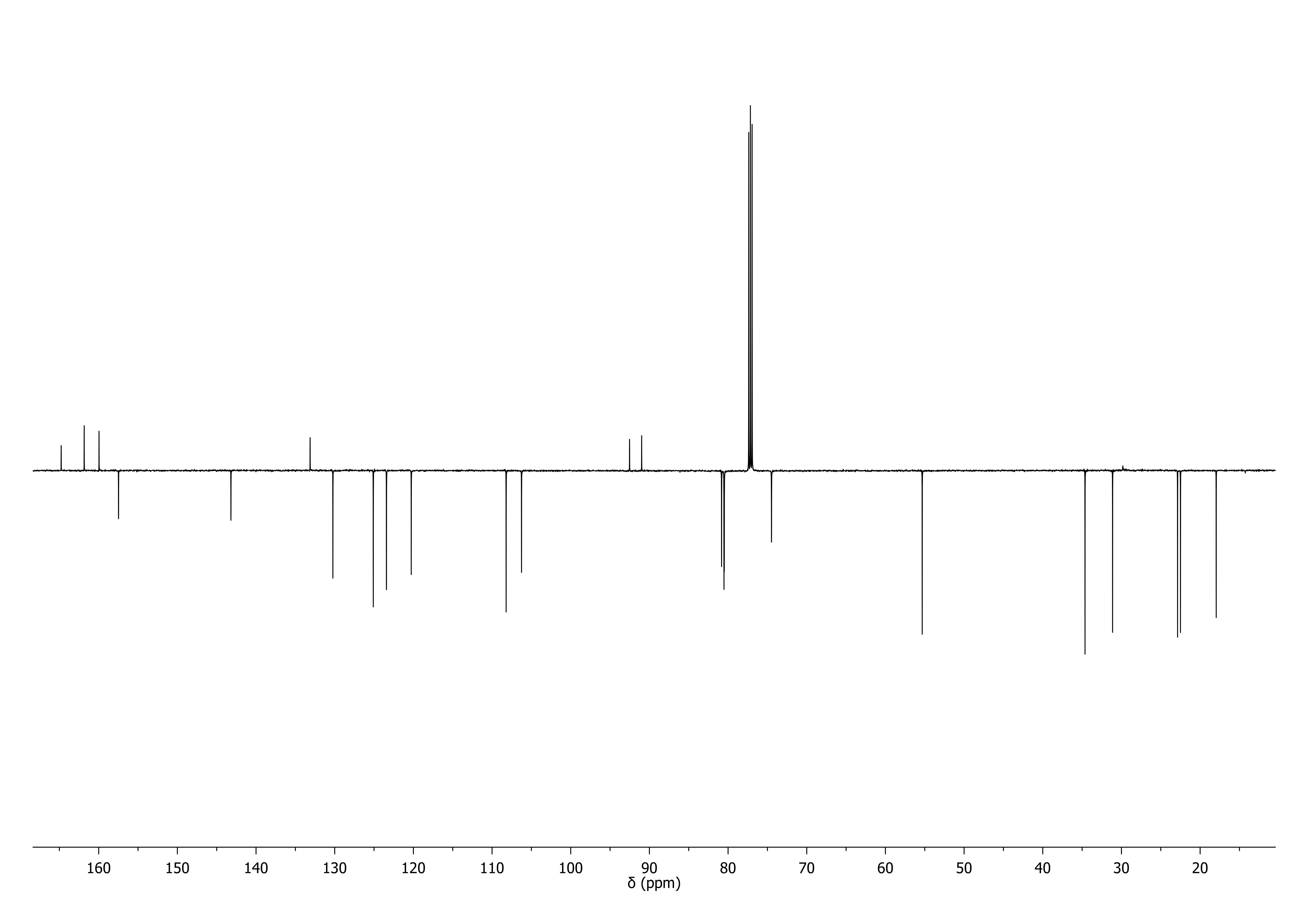


**Figure S10:** Top: Atom labelling and ^1^H-NMR spectrum of **4e**; Bottom: ^13^C-NMR spectrum of **4e**.

# Mass spectra

**Figure S11**: First and second row: Mass spectrum of **2a** (M-NO_3_)^+^; Third row: Calculated mass spectrum of **2a** (M-NO_3_)^+^.

**Figure S12:** First and second row: Mass spectrum of **2b** (M-NO_3_)^+^; Third row: Calculated mass spectrum of **2b** (M-NO_3_)^+^.

**Figure S13:** First and second row: Mass spectrum of **2c** (M-NO_3_)^+^; Third row: Calculated mass spectrum of **2c** (M-NO_3_)^+^.

**Figure S14:** First and second row: Mass spectrum of **2d** (M-NO_3_)^+^; Third row: Calculated mass spectrum of **2d** (M-NO_3_)^+^.

**Figure S15:** First and second row: Mass spectrum of **2e** (M-NO_3_)^+^; Third row: Calculated mass spectrum of **2e** (M-NO_3_)^+^.

**Figure S16**: First and second row: Mass spectrum of **4a** (M-NO_3_)^+^; Third row: Calculated mass spectrum of **4a** (M-NO_3_)^+^.

**Figure S17**: First and second row: Mass spectrum of **4b** (M-NO_3_)^+^; Third row: Calculated mass spectrum of **4b** (M-NO_3_)^+^.

**Figure S18:** First and second row: Mass spectrum of **4c** (M-NO_3_)^+^; Third row: Calculated mass spectrum of **4c** (M-NO_3_)^+^.

**Figure S19:** First and second row: Mass spectrum of **4d** (M-NO_3_)^+^; Third row: Calculated mass spectrum of **4d** (M-NO_3_)^+^.

**Figure S20:** First and second row: Mass spectrum of **4e** (M-NO_3_)^+^; Third row: Calculated mass spectrum of **4e** (M-NO_3_)^+^.

# X-ray diffraction data

A general description for data collection, structure solution and refinement are given in the main text. More details are given below.

**Figure S21**: Displacement ellipsoid plots^1^ (50% probability) of one monocationic complex of **3a** (left) and **4a** (right) in the asymmetric residue.


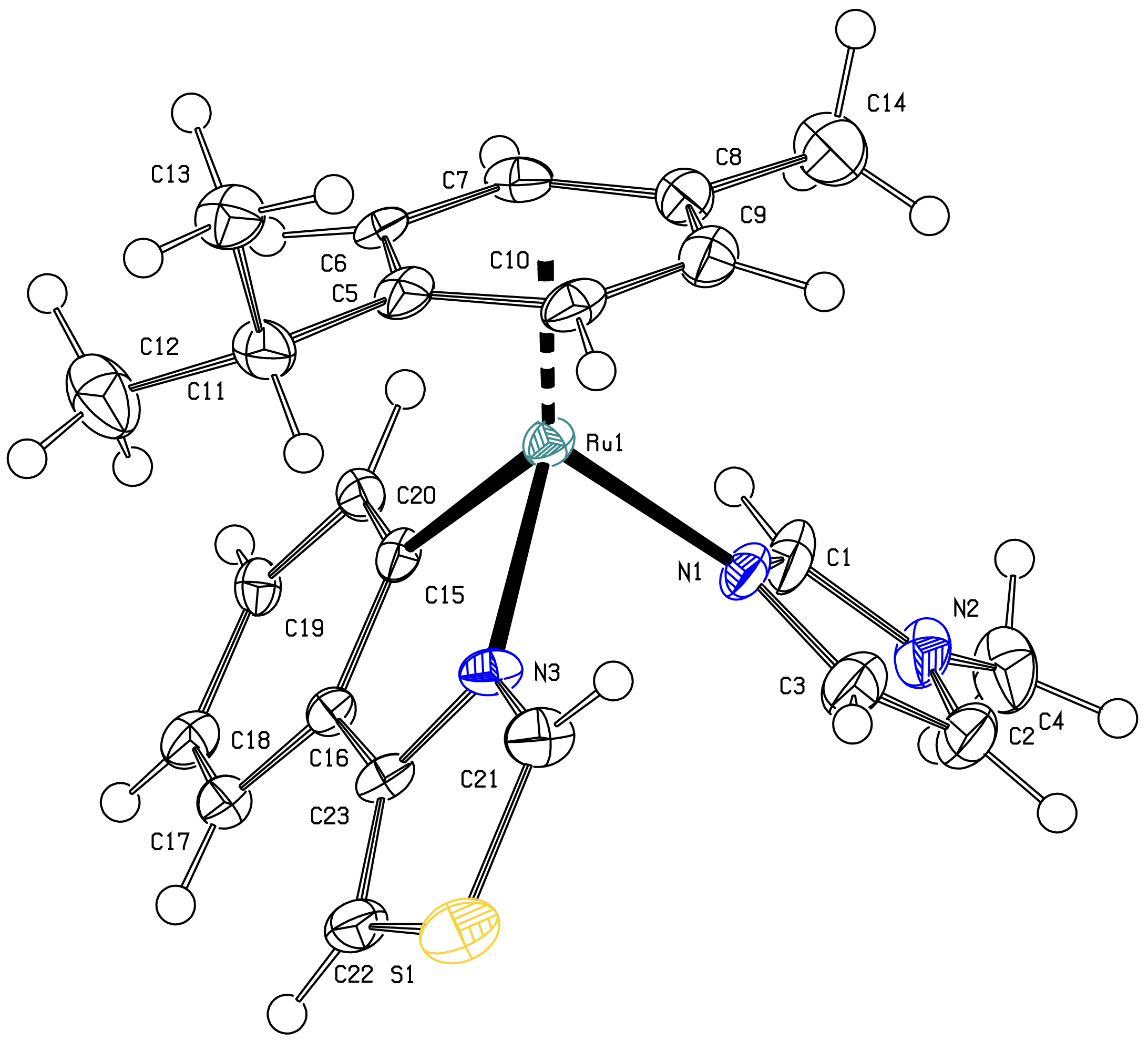

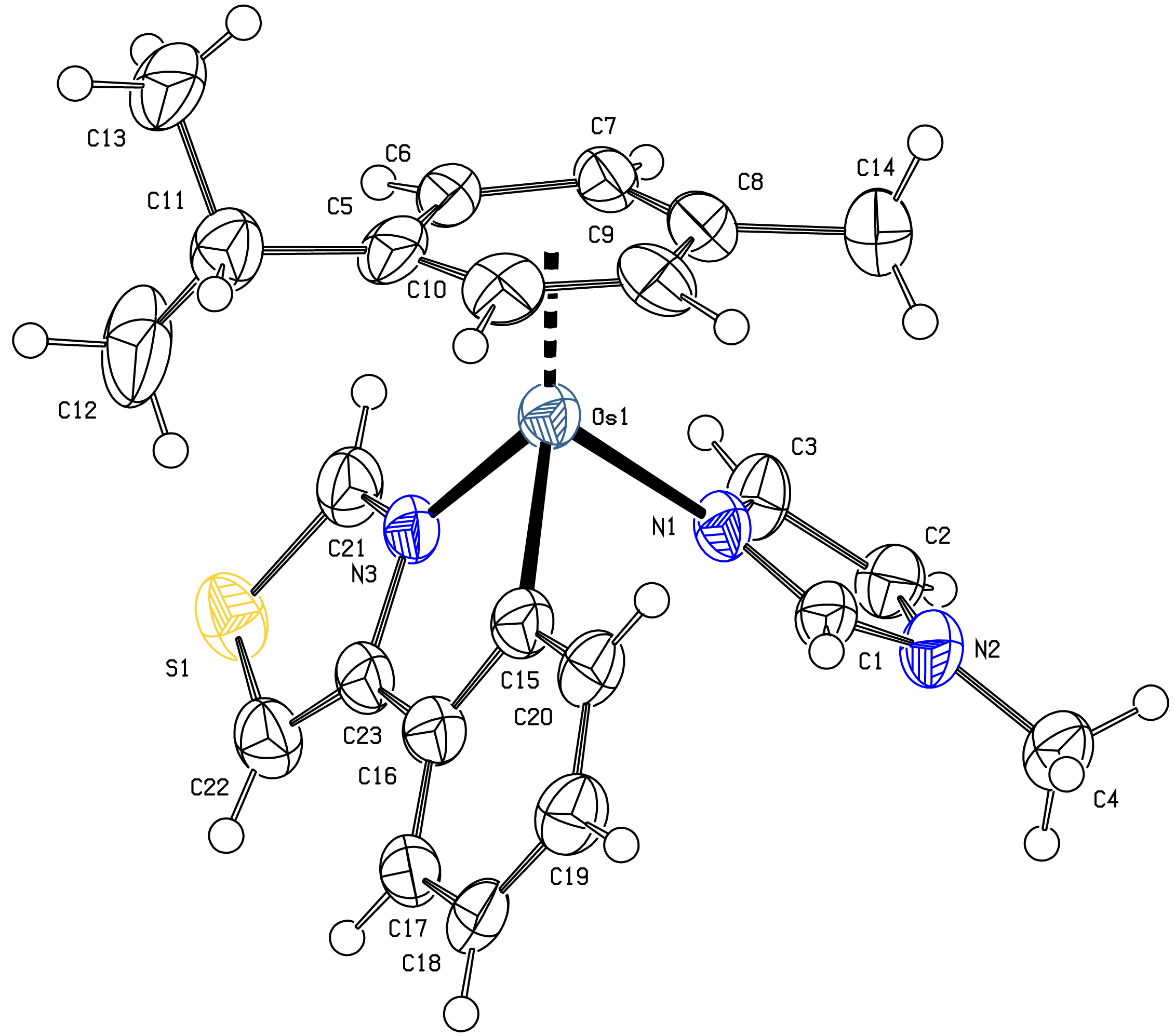


**Table S1**: Crystal data for **3a** and **4a**.

| Compound | **3a** | **4a** |
| --- | --- | --- |
| Empirical formula | C_23_H_27_N_4_O_3.5_RuS | C_23_H_27_N_4_O_3.5_OsS |
| Moiety formula | C_23_H_26_N_3_RuS, NO_3_, 0.5(H_2_O) | C_23_H_26_N_3_OsS, NO_3_, 0.5(H_2_O) |
| *M*_r_ / g mol^−1^ | 548.61 | 637.74 |
| Crystal habit | yellow block | yellow needle |
| Crystal size / mm^3^ | 0.12 x 0.08 x 0.08 | 0.10 x 0.03 x 0.03 |
| Crystal system | monoclinic | monoclinic |
| Space group (No.) | *P*2_1_ (4) | *Pc* (7) |
| *a* / Å | 9.9651(3) | 14.359(3) |
| *b* / Å | 23.7426(8) | 9.4275(19) |
| *c* / Å | 10.3470(3) | 34.424(7) |
| *β* / ° | 108.622(2) | 97.08(3) |
| *V* / Å^3^ | 2319.91(13) | 4624.5(16) |
| *Z*/*Z*’ | 4/2 | 8/4 |
| *D*_calc_ / g cm^−3^ | 1.571 | 1.832 |
| *µ* / mm^−1^ | 8.01 | 11.558 |
| *λ* / Å | 0.71073 | 1.54178 |
| sin(*θ*_max_)/*λ* / Å^−1^ | 0.704 | 0.626 |
| total/unique refl. | 43702/11627 | 79931/18338 |
| observed refl. | 8097 | 16832 |
| No. of parameters | 594 | 1118 |
| *R*_int_ | 0.0480 | 0.0577 |
| *R*_1_ (*I* > 2*σ*(*I*)) | 0.0449 | 0.0531 |
| *wR*_2_ (all data) | 0.1077 | 0.1425 |
| *S* (all data) | 0.974 | 1.052 |
| *ρ*_min_/*ρ*_max_ / e Å^−3^ | −2.459/0.989 | −1.870/2.528 |
| CCDC # | 2384986 | 2384985 |


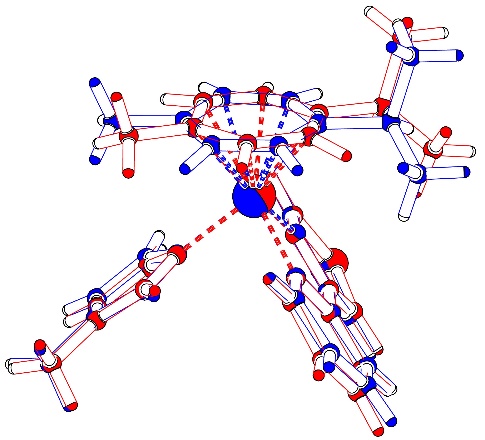


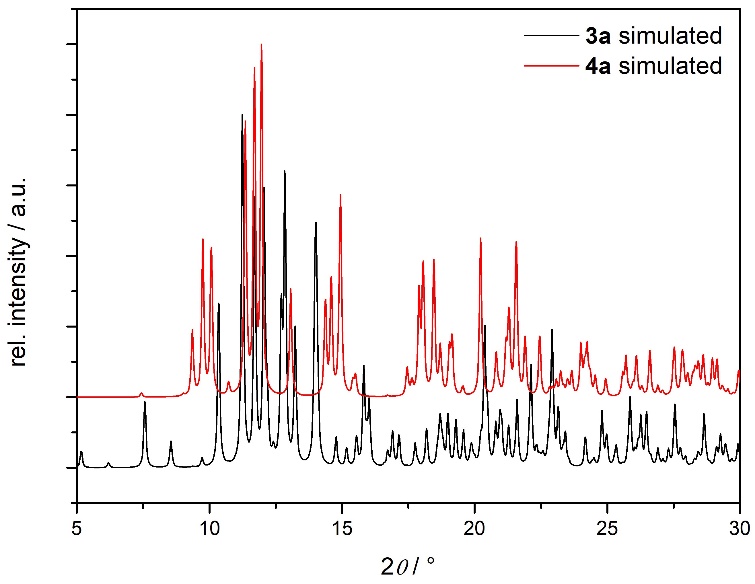


**Figure S22**: Overlay plot^1^ of the two symmetry independent complexes found in the solid-state structure of **3a**. The complexes exhibit rather similar coordination spheres, except the p-cymene ligand which shows a slight rotation of about 30 deg.

**Figure S23**: Comparison of the simulated powder patterns of **3a** and **4a**.

# Antiproliferative activity

**Figure S24**: Concentration-effect curves of ruthenium complexes **3a–3e** in A549 (top), CH1/PA-1 (center) and SW480 cells (bottom) relative to untreated controls (3c only shown in case of activity). Means ± standard deviations from at least three independent MTT assays.

**Figure S25:** Concentration-effect curves of osmium complexes **4a–4e** in A549 (top), CH1/PA-1 (center) and SW480 cells (bottom) relative to untreated controls (4c not shown due to inactivity). Means ± standard deviations from at least three independent MTT assays (exposure time: 96 h).

# Stability in aqueous solution

**Figure S26**: UV-vis absorption spectra of **3a** (a) and **3c** (b) at pH 1.0 followed in time. Dashed red spectra denote [Ru(p-cym)(Z)]^(2+n)^ (Z = Cl^-^ or H_2_O) (a) or [Ru(p-cym)(*N,C*)(Cl)] dissolved at the same conditions (b). Inserted figures show the changes of absorbance at 430 nm at the indicated pH values {c_complex_ = 100 μM (a), 90 μM (b); pH 1.0, 2.0 (HCl) or 7.4 (PBS); 25 °C}.

**Figure S27:** UV-vis absorption spectra of **3c** recorded at various pH values, the inserted figure shows the molar spectra of the species [Ru(p-cym)(*N,C*)(MeIm)]^+^ (MeIm-complex) and [Ru(p-cym)(N,C)(Z)]^n+^, Z = Cl^-^ or H_2_O (MeIm-free complex) {c_complex_ = 100 μM; *I* = 0.1 M KCl; sample incubation time: 3 days; 25 °C}.

**Figure S28:** UV-vis absorption spectra of **3a** recorded at various pH values, the inserted figure shows the changes of absorbance at 430 nm as a function of pH {c_complex_ = 157 μM; *I* = 0.1 M KCl; waiting time: 3 days; 25 °C}.

**Figure S29:** Distribution curves calculated for the dissociation of MeIm from **3c** at different pH values and complex concentrations of 0.1 – 100 μM {*I* = 0.1 M KCl; 25 °C}.

# Cellular Accumulation

**A**

**B**

**Figure S30:** A: Correlation between drug accumulation and calculated lipophilicity coefficients. B: Semi-logarithmic plot of cellular accumulation after 2 h exposure to compounds 3a–e vs. their IC50 values (96 h exposure) in SW480 cells.

# ROS investigation

**Figure S31**: Intracellular ROS levels in SW480 cells upon treatment with selected compounds, determined *via* the DCFH-DA assay in a time- and concentration-dependent approach. Tert-butyl hydroperoxide (TBHP) was used as positive control. Values were measured every 10 min over a total period of 2 h for each compound concentration. Means and SDs were calculated from three independent experiments.

# G-Quadruplex interaction

**Table S2**: 5’-3’ sequences. In dsDNA, Heg linker is [(-CH_2_-CH_2_-O-)_6_].

| **Oligo** | **Sequence** |
| --- | --- |
| dsDNA | FAM/TAT AGC TA-Heg-TATA GCT ATA/TAMRA |
| bcl2 | FAM/AGG GGC GGG CGC GGG AGG AAG GGG GCG GGA/TAMRA |
| h-Telo | FAM/GG GTT AGG GTT AGG GTT AGG G/TAMRA |
| hTERT | FAM/AGG GGA GGG GCT GGG AGG GC/TAMRA |
| Kit1 | FAM/AGG GAG GGC GCT GGG AGG AGG G/TAMRA |
| Kit2 | FAM/CGG GCG GGC GCG AGG GAG GGG/TAMRA |

**Figure S32**: FRET melting curves of the indicated oligonucleotides: a) dsDNA, b) bcl2, c) h-Telo, d) hTert, e) Kit1 and f) Kit2 when incubated with compounds **3a**, **3c** and **3d**. Buffer: 60 mM potassium cacodylate, pH 7.4.

# References

1 A. L. Spek, *Acta Crystallogr D Biol Crystallogr*, 2009, 65, 148–155.
